# Nanoparticles as Physically- and Biochemically-Tuned Drug Formulations for Cancers Therapy

**DOI:** 10.3390/cancers14102473

**Published:** 2022-05-17

**Authors:** Valentina Foglizzo, Serena Marchiò

**Affiliations:** 1 Department of Biochemistry and Molecular Biology, University of Miami Miller School of Medicine, Miami, FL 33136, USA; vxf221@miami.edu; 2 Department of Oncology, University of Torino, 10060 Candiolo, Italy; 3 Candiolo Cancer Institute, FPO-IRCCS, 10060 Candiolo, Italy

**Keywords:** nanoparticles, drug delivery, target therapy, cancer vaccine, cancer immunotherapy

## Abstract

**Simple Summary:**

Conventional antitumor drugs have limitations, including poor water solubility and lack of targeting capability, with consequent non-specific distribution, systemic toxicity, and low therapeutic index. Nanotechnology promises to overcome these drawbacks by exploiting the physical properties of diverse nanocarriers that can be linked to moieties with binding selectivity for cancer cells. The use of nanoparticles as therapeutic formulations allows a targeted delivery and a slow, controlled release of the drug(s), making them tunable modules for applications in precision medicine. In addition, nanoparticles are also being developed as cancer vaccines, offering an opportunity to increase both cellular and humoral immunity, thus providing a new weapon to beat cancer.

**Abstract:**

Malignant tumors originate from a combination of genetic alterations, which induce activation of oncogenes and inactivation of oncosuppressor genes, ultimately resulting in uncontrolled growth and neoplastic transformation. Chemotherapy prevents the abnormal proliferation of cancer cells, but it also affects the entire cellular network in the human body with heavy side effects. For this reason, the ultimate aim of cancer therapy remains to selectively kill cancer cells while sparing their normal counterparts. Nanoparticle formulations have the potential to achieve this aim by providing optimized drug delivery to a pathological site with minimal accumulation in healthy tissues. In this review, we will first describe the characteristics of recently developed nanoparticles and how their physical properties and targeting functionalization are exploited depending on their therapeutic payload, route of delivery, and tumor type. Second, we will analyze how nanoparticles can overcome multidrug resistance based on their ability to combine different therapies and targeting moieties within a single formulation. Finally, we will discuss how the implementation of these strategies has led to the generation of nanoparticle-based cancer vaccines as cutting-edge instruments for cancer immunotherapy.

## 1. Introduction

Cancer remains difficult to defeat despite the many efforts made by researchers to dissect the mechanisms of disease onset and progression. Driven by demographic changes and cumulative exposure to risk factors, the number of cancer patients is increasing, with an estimated 1.9 million new cases in in the United States in 2021 [1].

Surgery is the main curative option for most solid tumors, often accompanied by radiotherapy and/or chemotherapy. Radiation therapy uses X-rays to destroy cancer cells and is focused on the diseased area to avoid damaging healthy cells. It can be used (i) alone, if the tumor is sensitive to radiation, (ii) before surgery, to reduce the size of the tumor, and/or (iii) intraoperatively, to contain the risk of relapse. Chemotherapy uses cytotoxic drugs to kill cells that replicate rapidly. As such drugs do not distinguish between healthy and diseased tissues, their administration results in heavy side effects on rapidly remodeling sites such as mucous membranes, hair follicles, and blood cells. For a limited proportion of patients with breast and prostate cancer, hormone therapy may be used as an alternative to chemotherapy, with good efficacy and improved tolerability. More recently, biologically and molecularly targeted approaches have been developed that act specifically on cancer cells; for example, antibody–drug conjugates or tyrosine kinase inhibitors. The re-emergence of immunotherapy is also contributing to this relatively new repertoire of anticancer approaches, towards more focused and personalized therapies. An updated overview of current cancer management is detailed in the last Annual Report from the American Society of Clinical Oncology [2].

Due to the persistent limitations of current therapies, active research is ongoing for precise medical tools that can adapt to cancer type and stage, patient characteristics, genetic landscape, and so on. As early as 1907, Paul Ehrlich proposed the concept of a “magic bullet” to indicate a therapy that overcomes biological barriers, selectively targets cancerous tissues, and intelligently responds to the heterogeneous tumor microenvironment to achieve the on-demand release of therapeutic agents [3]. Fifty-two years later, Richard P. Feynman introduced the term “nanotechnology” in his famous talk ‘There’s plenty of room at the bottom’, meaning that much could be accomplished in physics and biology by manipulating nanosized objects [4]. The beginnings were slow, and we had to wait until the 1980s for the first report of a nanotechnology-based magic bullet—also known as a drug-carrying nanoparticle—for cancer therapy [5].

Nanocarriers for drug delivery are based on diverse materials, ranging from organic (lipid, protein, glycan) materials to synthetic polymers. A variety of compounds—chemicals, proteins, nucleic acids—may either be loaded into the nanocarrier or connected to its surface. The resulting nanoparticles may be further engineered to expose a targeting moiety—an antibody, a protein, a peptide, a sugar, or an aptamer. How these nanomodules are assembled depends on the desired application, type of drug, and site of action. Being aware of the manifold declinations of nanotechnology in cancer therapy, in this review, we will reconsider some fundamental concepts of nanocarrier-based drug delivery, with reference to the most recent applications.

## 2. Nanocarrier-Mediated Drug Delivery: Overcoming Solubility Issues, Improving Sustained Release, Favoring Both Cell Uptake and Tumor Delivery

### 2.1. In Vitro: Loading of Chemotherapeutic Drugs into Rationally-Designed Nanocarriers

Hydrophilic compounds are poorly taken up by cells because of their inability to cross the lipid-rich plasma membrane. Hydrophobic compounds tend to aggregate upon intravenous administration, leading to embolisms and local toxicity. Embedding the drug into a nanocarrier has long been regarded as a means to bypass solubility issues and improve chemical stability while keeping the drug physically separated from healthy tissues, thus minimizing off-target effects [6,7,8]. Examples of successful formulations are Doxil^®^—a polyethylene glycol (PEG)-decorated liposome encapsulating doxorubicin, first approved in 1995 by the Food and Drug Administration (FDA) for ovarian cancer and multiple myeloma—and Abraxane^®^—an albumin-bound nanoparticle loaded with paclitaxel, first approved by the FDA in 2005 for metastatic breast cancer [9]. Please refer to the review by Kumari et al. [10] for an extensive overview on drug encapsulation into nanoparticles, with reference to their chemistry and therapeutic implications.

Co-delivery of drugs that have opposing water solubilities is another challenging task that can be accomplished with proper engineering of the nanocarrier. On this basis, Barbalata et al. [11] manufactured liposomes co-encapsulating doxorubicin (hydrophilic) and simvastatin (hydrophobic) by following a rational design of lipid composition/drug concentration to achieve optimal drug loading and delivery to cancer cells. We believe that such a Quality by Design approach should be applied in the development of any new nanoparticle to provide a standard procedure that would likely accelerate approval for clinical applications. In a recent example, Lee et al. prepared water-oil-water double emulsion nanoparticles for the concomitant loading of doxorubicin and erlotinib (hydrophobic), a small-molecule inhibitor of epidermal growth factor receptors (EGFR, dysregulated in several cancer types) [12]. The nanoparticles were coated with rhamnolipid, a biosurfactant derived from *Pseudomonas aeruginosa*, to add stability and biocompatibility for intravenous injections, with promising results in a mouse model of squamous cell carcinoma.

### 2.2. In Vitro and In Vivo: Regulating Drug Release from the Nanoparticle

Liposomes are the most widely used nanocarriers, and several studies focus on modifying their structure to improve the sustained release of chemotherapies. Liposome composition can be finely tuned to obtain different release rates, as reported in a very recent study [13]. The authors prepared three types of doxorubicin-loaded, PEG-decorated liposomes, namely, (i) liposome-A (Doxil^®^) with hydrogenated soybean phosphatidylcholine (HSPC) as the lipid constituent and ammonium sulphate pH 5.5 in the internal phase; (ii) liposome-B, with cholesterol as the lipid constituent and sodium citrate pH 4.0 in the internal phase; and (iii) liposome-C, with 1,2-distearoylphoshphatidyl-ethanolamine-N-(methoxyPEG-2000) (DSPE-mPEG) as the lipid component and sodium citrate pH 5.0 in the internal phase. These liposomes provided a slow, medium, and fast release of doxorubicin, respectively. In the murine melanoma B16-BL6 model, liposome-B (medium release) achieved the most efficient drug delivery into tumor cells and had the best antitumor activity, thus outperforming the FDA-approved drug Doxil^®^.

Other types of nanocarriers are being tested in addition to liposomes. For example, a way to obtain nanoparticles with very slow release kinetics is described by Cardoso et al. [14]. The structure of their doxorubicin-carrying nanoparticles consists in a shell of poly(L-lactide) and a core of poly(D,L-lactide-coglycolide) (PLGA), a FDA-approved copolymer known to improve sustained release. The cumulative release of the drug lasted up to 96 days and was dependent on the thickness of the external shell, which is supposedly a parameter that can be easily tuned to obtain the desired release rate for a chemotherapy of choice. Liu et al. [15] investigated nanomicelles made of poly(D-glucosamine)/chitosan linked to a hydrophobic portion (deoxycholic acid) and a cationic portion (glycidyltrimethylammounium chloride). The components self-assembled into amphiphilic micelles of ~200 nm, which were loaded with doxorubicin. Drug release started at 2 h and increased slowly and constantly thereafter. Kalenichenko et al. [16] prepared calcium carbonate microcapsules with surface-absorbed alternate layers of polycation and polyanion moieties following different protocols. Doxorubicin inclusion was obtained either by coprecipitation during microcapsule synthesis or by diffusion into complete microcapsules. The deriving polyelectrolyte microcapsules allowed a slow, tunable (depending on the core/shell structure and number of layers) release of doxorubicin. In all three examples, validation was only performed in vitro; it would be significant to confirm the results in animal models in terms of drug circulation times and successful delivery in vivo.

### 2.3. In Vitro and In Vivo: Maximizing Cell Uptake and Intracellular Delivery

Cell uptake is dependent on nanocarrier shape and size. This feature is demonstrated, for example, in a recent study by Bai et al. [17]. The authors synthesized prodrug polymers based on PEG methyl ether methacrylate, three alternative cellulose backbones with different lengths, and camptothecin. In a water solution, these prodrugs self-assembled in rod-like micelles (also called “molecular bottlebrushes”) with different sizes and a cellular uptake from fast/high to slow/low for the shortest and longest length, respectively. Accordingly, in a mouse xenograft model of breast cancer, the shortest micelles showed deeper tissue penetration and an improved antitumor effect compared with the others.

To improve intracellular delivery, modern nanoparticles often include moieties with the capacity to respond to acidic pH (to favor endo/lysosomal escape) and/or reductive conditions (to trigger drug unloading at the intracellular conditions). Choi et al. [18] prepared self-assembled amphiphilic nanoparticles, including a drug-carrying hydrophobic portion and two hydrophilic portions. The latter were designed to function as sensors of acidic pH, with the inclusion of several disulfide bonds to be processed by intracellular reduced glutathione (GSH). Similarly, Ko et al. [19] describe amphiphilic micelles (“vitamicelles”), including vitamin E (hydrophobic) and vitamin B (hydrophilic) derivatives, along with portions responsive to both pH and GSH. These compounds were capable of self-assembly in a water solution, originating micelles that were loaded with doxorubicin, leading to rapid intracellular delivery and reduced drug efflux from cancer cells. Besides their improved therapeutic efficiency, vitamicelles are interesting in the light of potential clinical applications because of their biocompatible and biodegradable composition. Finally, an innovative strategy exploits hollow nanoparticles with an internal “artificial cytosol” that resembles the intracellular milieu. Zong et al. [20] prepared distearoyl-sn-glycero-3-phosphocholine (DSPC)/cholesterol vesicles, including an artificial cytosol based on agarose and sucrose in a 1:9 ratio to reproduce the viscosity of natural cytosol. Doxorubicin-loaded vesicles were tested in cytotoxicity assays in vitro, demonstrating a 28.7-fold increased efficiency over the free drug.

The nanoparticles discussed in these paragraphs are summarized in Table 1.

### 2.4. In Vivo: Optimizing Biodistribution and Pharmacokinetics

The biodistribution of anti-cancer drugs depends on their physicochemical properties. In addition, some drugs are susceptible to enzymatic cleavage and/or hydrolytic degradation, with consequent short half-lives and scarce bioavailability. Finally, most drugs have inadequate pharmacokinetic profiles due to poor penetration into tumor tissues, clearance by the reticuloendothelial system, and renal excretion. Including the drug in rationally designed nanoparticles would not only protect them from degradation, but also improve their pharmacokinetic profile by allowing longer circulation times. A common solution to render nanoparticles invisible to the reticuloendothelial system is to decorate their surfaces with PEG, a broadly employed approach that has been known for the past 30 years [21] and is nowadays applied in most liposome preparations.

Once the drug is loaded into a nanoparticle and allowed to circulate for a sufficient time, it needs to be released precisely at the tumor site to achieve maximum efficiency with minimal side toxicity. A way to achieve this aim involves the induction of in situ hyperthermia to destabilize the nanoparticle and provoke an on-demand release of its content. A nanoparticle with this mechanism of action, lyso-thermosensitive liposomal doxorubicin (ThermoDox^®^), is in the phase of advanced testing in clinical trials. The liposome carrier is a mixture of 1,2-dipalmitoyl-sn-glycero-3-phosphocholine (DPPC):DSPE-mPEG:1-stearoyl-2-hydroxy-sn-glycero-3-phosphocholine (MSPC), and becomes unstable at temperatures >39.5 °C. In animal models, a 40 min warm-up of 40–45 °C leads to a sixfold higher intratumoral concentration of doxorubicin, and a 4.4 ratio of drug uptake by the tumor compared with the heart [22]. Based on these promising results, clinical trials have been designed, culminating in a Phase III study of ThermoDox^®^ in combination with radiofrequency ablation for primary liver cancer (the OPTIMA Study, NCT02112656). The study was discontinued because the endpoint was not reached, and other trials are being planned with different dosing and heating schedules. In their excellent review, Borys and Dewhirst examine all stages of ThermoDox^®^ development, providing a critical discussion of the unsuccessful strategies adopted, as well as suggestions on how to improve the translation of nanomedicines to clinical practice [23].

Other approaches combine heat-mediated nanoparticle disaggregation with a therapeutic use of hyperthermia. For example, in a very recent work [24], the authors embedded doxorubicin and carbonyl manganese-functionalized cupric sulfide particles into a shell of the heat-responsive amphiphilic copolymer polycaprolactone-Diels-Alder-PEG. Cupric sulfide particles act as photothermal reagents and heat up when irradiated with near infrared light (NIR), causing the disruption of the shell and the release of both doxorubicin and carbon monoxide (a chemosensitizer). In addition, the small sized cupric sulfide particles, once released promptly, enter the tumor mass, thus providing a means for hyperthermia treatments. As well as encouraging preliminary results obtained both in vitro and in animal models, each component has proven their efficacy as a single agent; therefore, their combination is expected to provide additive/synergistic effects.

Additional ways of circumventing biodistribution and pharmacokinetic issues are pertinent to passive and active targeting; therefore, they will be discussed in detail in subsequent paragraphs.

## 3. Drug Delivery Systems Based on Passive and Active Targeting

Passive targeting consists of the accumulation of the nanoparticle at the diseased site as a result of the body’s natural response to the physicochemical characteristics of the nanocarrier and/or the embedded drug so that the tumor becomes a favored site for drug delivery. Active targeting implies the addition of one or more ligands on the surface of the nanoparticle. By selectively interacting with cancer cell-specific (overexpressed or unique) receptors, the ligand moiety promotes specific binding of the nanoparticle and internalization of its payload.

### 3.1. Passive Targeting

#### 3.1.1. Enhanced Permeability and Retention (EPR) Effect and Transendothelial Migration

Tumor vasculature is characterized by the presence of leaky vessels, through which macromolecules extravasate and accumulate, a phenomenon described by Matsumura and Maeda in 1986 [25] as the EPR effect. A key feature for macromolecular compounds to exploit the EPR effect is size. In a very recent work, Gao et al. prepared nanoparticles based on an mPEG-caprolactone copolymer with hydrodynamic diameters between 38 and 135 nm [26]. These nanoparticles were injected into nonobese diabetic/severe combined immunodeficiency (NOD/SCID) mice, and their blood concentration was evaluated after 48 h, showing that the smaller the nanoparticle, the longer its circulation time and the lower its clearance. As a result of these pharmacokinetic parameters, small-sized nanoparticles accumulated at tumor sites and released higher payloads of doxorubicin in vivo. In this study, the optimal nanoparticle size was set at 62 nm.

Different nanoparticles have been designed to exploit the EPR effect, such as micelles, liposomes, and various natural or synthetic polymers, for applications in gene therapy, molecular imaging, and drug delivery. More specifically, those approved for cancer treatment are described in a review by Tracey et al. [9] and include (i) liposomes for the delivery of doxorubicin (Doxil^®^, Myocet^®^), daunorubicin (DanuoXome^®^), cytarabine (DepoCyt^®^), mifamurtide (MEPACT^®^), vincristine sulphate ((Marqibo^®^), irinotecan (Onivyde^®^) or a combination of cytarabine and daunorubicin (Vyxeos^®^), (ii) albumin-based nanoparticles (Abraxane^®^), and (iii) lipid micelles (Apealea^®^) for the delivery of paclitaxel. Despite early access for clinical practice use, overall, these approaches have only had partial success. The EPR effect is limited by different characteristics of the tumor, namely, (i) unevenness of vascular permeability, (ii) heterogeneous stroma, (iii) presence of necrotic areas, and (iv) high interstitial pressure. Several comprehensive reviews have dealt with the EPR topic; we found those from Maeda et al. [27], Golombek et al. [28], and Subhan et al. [29] of particular interest. A special issue of *Theranostics* includes 24 papers and reviews on the connections between nanoparticles and the EPR effect [30].

Sindhwani et al.—quite unexpectedly—found that up to 97% of nanoparticles access the tumor tissue by transendothelial migration and not via the EPR effect [31]. These findings may help design next generation nanoparticles for passive drug targeting that include not only an optimal size (10–200 nm), but also a proper charge for adhesion to the surface of endothelial cells. It has long been known that the circulation-accessible side of blood vessels bears a substantial negative charge, so positively charged macromolecules are expected to be promptly captured. In a pioneering study [32], doxorubicin-embedding cationic liposomes were prepared by adding 1,2-dioleoyl-3-trimethylammonium-propane to a lipid mixture that included 1,2-dioleoyl-sn-glycero-3-phosphocholine, cholesterol, and DSPE-PEG. Compared with their neutral counterpart, positively-charged liposomes showed decreased uptake by the spleen and blood, and increased accumulation in tumor blood vessels, although there was no overall increment in tumor uptake.

#### 3.1.2. Exploiting Biochemical Properties of the Tumor Microenvironment: Nanocarrier-Loaded Drugs

The latest prototype nanoparticles display additional portions responsive to the chemical properties of the tumor microenvironment, namely, mildly low pH (acidification is a common effect of tumor cell metabolism), reactive oxygen species (ROS, produced by cancer cells in metabolic stress conditions), and/or reductive conditions (high levels of GSH are present in the tumor circulation). This approach is expected to increase drug release at the tumor site, thus potentiating the EPR effect.

Xu et al. [33] designed amphiphilic nanocarriers based on hydroxyethyl starch, modified by imidazole (hydrophilic portion) and cholesterol (hydrophobic portion). In the process of self-assembly, these nanoparticles encapsulated doxorubicin, which was slowly released in acidic conditions, as verified both in vitro and in vivo in a subcutaneous tumor model. Aziz et al. [34] prepared and thoroughly characterized albumin nanoparticles carrying doxorubicin, which were spheroidal in shape and with diverse sizes from 45 to 250 nm. In these nanoparticles, assembly occurred via electrostatic interactions between a protonated amine group on doxorubicin and negative charges on albumin. At the physiological pH (7.4), 52.0% of the drug was released in the first 12 h and another 19.4% was released over the successive 24 h; at lower pH (6.4), drug release increased to 63.5% in the first 12 h and 30.0% over the successive 24 h.

In another work [35], PEGylated nanoparticles based on calcium carbonate were conjugated to doxorubicin. Calcium carbonate becomes unstable in acidic conditions, and this feature was exploited to control drug release. At the neutral pH, 20% of the doxorubicin content was released over 3 h; this value increased up to ~40% at pH 6.0 (extracellular microenvironment) and ~80% at pH 5.0 (intracellular lysosomal compartment).

As a further example, nanocapsules were prepared using the self-assembly of an amphiphilic polymer consisting of a hydrophilic stretch (series of carboxyl groups) and a hydrophobic portion (ferrocenylmethyl methacrylate) [36]. Ferrocene becomes oxidated in the presence of ROS, with the formation of a ferrocenium cation; the change in polarity causes the disaggregation of the nanocapsule shell. By tuning the solvent polarity in the process of assembly, the authors produced five renderings of nanocapsules with different sizes (71–200 nm) and responsiveness to ROS (different surface charges), which were loaded with paclitaxel. In vitro, differential drug release was achieved in the absence (17% in the first 2 h, 27% within 24 h) or in the presence (54% in the first 2 h, 85% within 24 h) of H_2_O_2_.

Multiple agents can be included in this type of biochemically tuned nanoparticle to potentiate their efficacy. In a recent study, doxorubicin and paclitaxel were coloaded into single-walled carbon nanotubes [37] grafted with dimethyl acrylamide–trimethyl chitosan. At the physiological pH, doxorubicin has a net positive charge that allows an electrostatic bond with the negatively charged chitosan polymer, thus favoring drug loading. At pH 5.5, these charges are lost, and drug release takes place. Instead, paclitaxel is bound by interactions of the Van der Waals type and released at an acidic pH due to degradation and deformation of the polymer. Although theoretically interesting, this approach has not been tested on cell lines or animal models, so its potential can only be inferred.

#### 3.1.3. Exploiting Biochemical Properties of the Tumor Microenvironment: Prodrug-Based Nanoparticles

Alternative strategies use the drug itself as a structural component of the nanoparticle (in the form of prodrug). For example, poly(doxorubicin)-PEG polymers with a drug content of ~75% were self-assembled in spherical nanoparticles of 135 nm. A negligible burst release of doxorubicin was observed at the physiological pH, whereas sustained drug release was activated at a lower pH, reaching ~50% at a of pH 5.0 over 60 min [38].

Wang et al. [39] conjugated cisplatin to PEG via two functionally distinct portions (i.e., adipic acid dihydrazide (responsive to pH) and 3,3′-disulfanediyldipropionic acid (responsive to GSH via the disulfide bond)). From this prodrug, nanoparticles were prepared with the addition of glyceryl monostearate, soybean lecithin, and paclitaxel, obtaining a final size of ~115 nm and a drug loading efficiency of >80%. In vitro, the release of both cisplatin and paclitaxel was increased by a low pH and high GSH, reaching a maximum of ~90% at pH 5.0 and 10 mM GSH. The nanoparticles were efficiently taken up by tumor cells in vitro, with consequent cytotoxic effects, and showed encouraging antitumor activity in a mouse model of lung cancer.

Several works report the application of paclitaxel as a model chemotherapeutic drug. In a study by Lu et al. [40], paclitaxel dimers were prepared with different linkers (dicarbide, disulfide, or diselenide bonds) and carriers (Pluronic^®^ F127, DSPE-PEG, human serum albumin, or Fe-tannic acid). Testing the derived nanoparticles revealed the optimal performance of diselenide bond-including paclitaxel dimers (in terms of response to reductive conditions) included in the Pluronic^®^ F127 nanocarrier (in terms of accumulation at the tumor site and therapeutic effect in a xenograft model of triple-negative breast cancer). Similarly, Kanwal et al. [41] synthesized a conjugate of paclitaxel and dextran via a disulfide bond linker. The polymers self-assembled, giving nanoparticles of 110 ± 34 nm, which were sensitive to increasing concentrations of a reducing agent, as evaluated in vitro on two cancer cell models. Zou et al. [42] linked paclitaxel and tetramethylpyrazine via a disulfide bond, thus obtaining an amphiphilic conjugate. Spherical nanoparticles of 152 nm were prepared from the conjugate by self-assembly in a water solution. These nanoparticles were sensitive to reductive conditions; in the presence of GSH, the structure disassembled with destruction of the disulfide bond and release of the drug. The authors provide results in support of the efficacy of this system both in vitro and in a tumor xenograft model.

The nanoparticles that exploit biochemical features of the tumor microenvironment described above are recapitulated in Table 2.

#### 3.1.4. Exploiting Biochemical Properties of the Tumor Microenvironment: Multimodal Nanoparticles

The delivery of chemotherapeutic drugs may be accompanied by at least one other function, such as photodynamic or photothermal therapy.

In one example [43], a strategy was designed to integrate photodynamic and chemodynamic therapy in a pH-responsive system. This strategy was based on upconversion nanoparticles (capable of emitting UV/visible light when excited with NIR light) (i) covered by albumin, (ii) associated with both the photosensitizer chlorine 6 (to produce ROS when activated by UV light—photodynamic therapy) and Fe^2+^ (Fenton reagent, to produce ROS in the presence of H_2_O_2_—chemodynamic therapy), and (iii) loaded with doxorubicin (to induce the production of H_2_O_2_—chemodynamic therapy in addition to its cytotoxic effect). When at the physiological pH, the nanoparticles were stable, whereas at pH 5.6 they released >35% of the doxorubicin payload, leading to an increased concentration of H_2_O_2_ and triggering the chemodynamic effect. Similarly, Luo et al. synthesized doxorubicin-containing block copolymers that self-assembled in nanoparticles of 122.6 ± 5.5 nm [44]. These polymers included disulfide bonds to respond to intracellular GSH with the release of doxorubicin. Chlorin 6 was also included for concomitant photodynamic therapy upon NIR irradiation.

In another example [45], a “nano-donut” formulation of doxorubicin was prepared to combine magnetic resonance imaging (MRI) with photothermal and chemodynamic therapy. These donut-shaped nanoparticles were composed of Prussian Blue (photothermal agent), combined with Cu^2+^ and Mn^2+^ ions, and they reacted with molybdenum salt to achieve the final structure. At the tumor site, the presence of H_2_O_2_ caused decomposition of the nanoparticle with consequent production of cytotoxic ROS, release of doxorubicin, and higher photothermal efficiency of Prussian Blue under NIR irradiation. The authors tested the nano-donuts both in vitro and in vivo, with promising results.

Wang et al. [46] prepared a nanocarrier based on polyallylamine-PEG-dimethylacrylamide/poly(ethylene imine) (PEI)-poly(ε-caprolactone) polymers coloaded with docetaxel and a photosensitizer (IR825, a NIR small-molecule cyanine dye for photothermal therapy). The deriving nanoparticles had a size of ~130–145 nm. Mild acidic conditions (pH 6.8) caused charge reversal, disruption of the nanoparticle shell, and drug release. These nanoparticles proved more efficient in killing tumor cells compared with their non-charge-reversal counterparts both in vitro and in vivo in a xenograft model of breast cancer.

Yu et al. [47] used a semiconducting polymer as a photothermal agent and doxorubicin as a chemotherapeutic drug. Modified semiconducting polymers self-assembled with a PEG-doxorubicin conjugate, including a pH-responsive portion to give nanoparticles of ~50 nm. The photothermal effect was activated by irradiation with low-power NIR light for 5 min, enhancing doxorubicin release at an acidic pH and activating therapeutic hyperthermia both in vitro and in vivo.

Finally, dendrimer/lentinan-based nanoparticles were studied for concomitant paclitaxel-based chemotherapy and photodynamic therapy [48]. The dendrimer was decorated with phenylboronic acid (a lectin mimic that binds sialic acid residues on the cell surface) and included a photosensitizer bound to lipoic acid (GSH-responsive moiety). The components self-assembled to give nanoparticles of ~130 nm, which were responsive to the acidic pH in terms of lower structural stability and increased drug release.

These multimodal nanoparticles are summarized in Table 3.

#### 3.1.5. Exploiting Specific Molecular Features of the Tumor Microenvironment

In some strategies, the EPR effect is implemented by exploiting molecular mechanisms unique of, or enriched in, the tumor microenvironment.

A liposome that reverts its charge from negative to positive in the presence of matrix metalloproteinase 9 (MMP9, upregulated in several cancer types) has been recently described [49]. This liposome incorporated an amphiphilic molecule containing oleic acid (hydrophobic portion) and a glutamate-rich stretch (negative charges, hydrophilic portion) linked to a peptide substrate of MMP9. In the presence of MMP9, the poly-glutamate portion was released, and the liposome acquired a positive charge leading to increased endocytosis. This property was validated in MMP9-expressing xenografted models, where the diagnostic (cyanine dye) or therapeutic (doxorubicin) payload was efficiently and specifically delivered.

In another work, Chen et al. [50] prepared liposomes responsive to MMP2, which is another extracellular enzyme enriched in the tumor microenvironment. An anti-CD147 antibody was linked to the liposome surface via a peptide substrate of MMP2 and paclitaxel was loaded into the core. In the presence of MMP2, the antibody was released, and induced the conversion of M2 (pro-tumoral) into M1 (anti-tumoral) macrophages. The release of paclitaxel provided concomitant cancer cell cytotoxicity and inhibition of metastasis, as demonstrated in a mouse model of triple-negative breast cancer.

**Figure 1:**
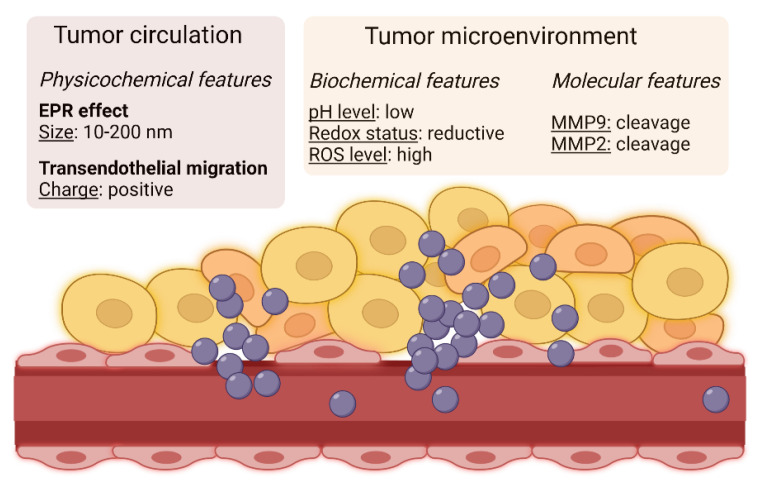
Passive targeting of circulating macromolecules is based on the EPR effect (entry and accumulation through openings between endothelial cells) and on transendothelial migration. These mechanisms are maximized for nanoparticles with proper size and charge. In addition, the tumor microenvironment holds chemical (pH level, redox status, production of ROS) and molecular (presence of specific enzymes) characteristics that can be exploited to specifically activate drug delivery by the inclusion of responsive elements in the nanoparticle structure. Created with BioRender.com.

The most relevant parameters that can be implemented in the design of nanoparticles for passive targeting of cancer are schematized in Figure 1.

### 3.2. Active Targeting: Antibodies and Antibody Fragments as Targeting Moieties

#### 3.2.1. Antibodies

A way to direct a nanoparticle to a tumor mass is to decorate its surface with an antibody that works against a tumor-specific plasma membrane protein. Immunoglobulins G (IgGs) hold optimal properties for such targeted approaches, namely, high affinity/specificity of binding and ease of chemical manipulation/linking to the nanoparticle shell. Therapeutic antibodies produced in heterologous hosts—as has been common practice since the 1950s—lead to the activation of immune responses and allergic reactions. Current approaches, instead, include humanized or human IgGs, which are engineered to improve affinity and reduce immunogenicity, while keeping their antigen specificity.

In a pioneering study, Torchilin’s group prepared micelles based on PEG-phosphatidylethanolamine and decorated with a cancer-specific anti-nucleosome monoclonal antibody (mAb) [51]. The derived immunomicelles targeted different cancer cells in vitro and in vivo, and efficiently delivered taxol to mouse models of Lewis lung carcinoma, with enhanced tumor inhibition compared with the free drug, and to untargeted micelles. A recent work from the same group describes liposomes functionalized with 2C5, a mAb against cancer overexpressed nucleolin, for the codelivery of paclitaxel and salinomycin (to target both bulk cancer cells and cancer stem cells). These immunoliposomes, with sizes ranging between 170–220 nm and which have a negative charge, were efficiently loaded with both drugs at a 1:1 ratio. Their therapeutic properties were validated in vitro in two models of breast cancer [52].

Many other studies originated from this starting point, with the aim of optimizing the antibody-mediated targeting of cancer cells and/or cancer-associated cells. Please refer to the excellent reviews by Petrilli et al. and Silvestre et al. [53,54] for a complete overview of immunoconjugates for cancer targeting (including antibody-functionalized nanoparticles) and nanosystem-included antibodies (or biosimilars), respectively.

Antibodies already approved for cancer therapy are often used as targeting moieties. For example, trastuzumab (an anti-human epidermal growth factor receptor 2, HER2, mAb used in breast cancer management) was conjugated on the surface of nanorods (size ~95 × 500 nm), including the chemotherapeutic drug paclitaxel, with a molar ratio paclitaxel:trastuzumab of 682:1 [55]. The nanorods were tested in vitro for their specificity on two HER2-expressing breast cancer cells lines, in comparison with a negative cell line, observing the specific induction of apoptosis (>80% in target cells) with a synergistic effect. In another work, trastuzumab-decorated nanoparticles were prepared with gold nanorods in a core of perfluorohexane and paclitaxel in a shell of PLGA-PEG [56]. The drug content could be released upon laser irradiation and the gold nanorods provided the nanoparticles with additional diagnostic properties. To precisely control coupling of trastuzumab to the nanoparticle surface, Escareno et al. [57] proposed a microfluidic-based system. The authors used a Y-shaped microreactor to covalently bind trastuzumab to doxorubicin-loaded chitosan/PLGA nanoparticles. In this way, they obtained nanoparticles with better performance in vitro, in terms of controlled size, drug release kinetics, and specific cancer cell uptake/killing, compared with nanoparticles functionalized with a bulk approach. Other anti-HER2 antibodies have been developed for similar purposes. A commercially available mAb (clone 7H3L20) was linked to the surface of gemcitabine-loaded fucoidan/chitosan nanoparticles [58]. The resulting nanoparticles had a size of ~160 nm and a positive charge; their capacity to specifically target HER2-expressing breast cancer cells was confirmed both in vitro and in vivo, where treatment with the targeted nano-formulation led to both the reduction of primary tumor growth and inhibition of lung metastasis in an orthotopic mouse model of breast cancer.

Among other clinically approved antibodies, the EGFR-specific mAb cetuximab has been largely exploited in the functionalization of targeted nanomedicines. El Hallal et al. [59] describe the in vitro characterization of gold nanoparticles of different sizes (25, 40, 60, and 80 nm) decorated with cetuximab. In cytotoxic assays against colon cancer cell lines, the 60 nm nanoparticles proved to be the most effective compared with the other formulations and free cetuximab. In another work, an anti-EGFR mAb was linked to the surface of PLGA-PEG nanoparticles carrying the chemotherapeutic 5-FU [60]. The specificity of these 252.3 nm negatively charged nanoparticles was confirmed in vitro in a cytotoxicity assay on a colorectal cancer cell line.

A variety of additional mAbs and corresponding targets have been explored as well. Castro et al. [61] describe 200 nm positively charged chitosan-PEG-Poloxamer 188 nanocapsules embedding docetaxel in the oily core, which are surface-decorated with Chi-Tn, a mAb specific for the Tn antigen O-[2-(Acetylamino)-2-deoxy-α-D-galactopyranosyl]-L-serine (a glycosidic structure characteristically present in carcinomas). Attachment of the mAb on the nanocapsule surface was achieved via a biotin–avidin system. The nanocapsules exhibited a sustained release of docetaxel, which was dependent on pH, being increased in acidic conditions.

Navarro-Marchal et al. [62] synthesized nanocapsules with (i) a core of olive oil loaded with paclitaxel and (ii) a shell of phosphatidylcholine, Pluronic^®^ F68, and deoxycholic acid. The nanoparticles were functionalized with a mAb against CD44, a cell surface marker of stem cells [63], and were capable of efficiently binding and killing pancreatic cancer stem cells in vitro and targeting an orthotopic model of pancreatic cancer in vivo.

Pindiprolu at al. [64] prepared solid lipid nanoparticles incorporating N-[N-(3,5-Difluorophenacetyl)-l-alanyl]-S-phenylglycine T butyl ester, an inhibitor of γ-secretase (a surface enzyme responsible for the cleavage of different proteins, including Notch, in cancer progression) [65]. Conjugation with a mAb against death receptor-5 (DR5) resulted in nanoparticles of 187 ± 0.98 nm and a positive charge, specifically for breast cancer cells, as evaluated in vitro and confirmed in vivo in a chemically induced tumor model.

Sortilin, a protein associated with the aggressiveness of different types of cancer, including breast, ovarian, and pancreatic, is an additional target for antibody-directed nanomedicines. The sortilin-specific mAb 2D8-E3 was conjugated onto halloysite nanotubes carrying docetaxel and including gold nanoparticles [66]. In this system, drug release is favored by the multilayered structure of the nanotubes, which responds to a low pH and high temperature, and by the induction of a photothermal effect by irradiation of the gold particles. A strong cytotoxic effect was observed in vitro in a cell line of ovarian carcinoma.

Finally, Tian et al. [67] describe a prodrug based on α-squaric acid ethylester-PEG-2-ethylbutoxy-2-oxo-1,3,2-dioxaphospholane copolymer linked to camptothecin via a disulfide bond. The derived nanoparticles were functionalized with a mAb against CD147, a transmembrane glycoprotein overexpressed in epithelial cancer cells. These nanoparticles were efficiently recognized and internalized by liver cancer cells in vitro.

Model nanoparticles that use mAbs as targeting moieties are reported in Table 4.

#### 3.2.2. Antibody Fragments

Antibodies are large in size (~150 kDa), which limits their density on the nanocarrier. Alternative strategies use antibody fragments to achieve the same specificity in a much smaller molecule. A broad variety of fragments have been exploited, including Fab, F(ab’)2, diabodies, single-chain Fv fragments (scFv), minibodies, nanobodies, and their combinations. Please refer to the excellent publication by Bates et al. for a detailed review on this topic [68].

Many antibody fragments have been introduced in the clinical field as single agents [69] and some of them are being investigated as targeting moieties coupled to different nanoparticles. One of the first applications of a F(ab’)_2_ was described in 1980 [70]. In this work, each lipid vesicle was decorated with 143 molecules of rabbit antibody fragments against human erythrocytes, leading to ~8000 F(ab’)_2_-decorated vesicles selectively bound per target cell (200-fold higher than those bound to control cells).

Among the most recent applications, Ni et al. [71] functionalized nanoparticles with the scFv of trastuzumab to deliver the maytansine analogue DM1 (MTC-100038) to HER2-positive cells. In vitro, uptake of the targeted formulation was increased, and viability of breast cancer cells was decreased, compared with the untargeted counterpart. Accordingly, targeted nanoparticles accumulated at tumor sites in vivo and exerted a more efficacious antitumor effect with an inhibition rate of 70%. In another work, mesoporous silica nanoparticles were modified with a poly(N-isopropylacrylamide-comethacrylic acid) polymer and an anti-HER2 scFv, which was covalently linked on their surface [72]. Doxorubicin release was dependent on pH, being 12.2% at pH 7.4, 21.8% at pH 6.5 (mild acidity of the tumor microenvironment), and 43.1% at pH 5.0 (acidity of the lysosomal compartment). It was also dependent on temperature, being 9.0% at 25 °C, 27.6% at 37 °C, and 38.2% at 41 °C. This controlled release was accompanied by a specific uptake in HER2-positive breast cancer cells, compared with nontumor cells. In vivo, the scFv-targeted/doxorubicin-loaded nanoparticles specifically accumulated at the tumor site, with only residual uptake by normal organs after 24 h. Treatment with these nanoparticles led to tumor regression in mice bearing subcutaneous xenografts of human breast cancer cells, with an efficacy that exceeded that of free doxorubicin or untargeted doxorubicin-loaded nanoparticles.

Lourenco et al. [73] set up a protocol for the site-oriented linking of a CD44v6 half-antibody fragment (obtained via chemical reduction of the full mAb) to polystyrene nanoparticles. The resultant 200 nm nanoparticles preferentially interacted with CD44v6-expressing gastric cancer cells in vitro, although binding (1.65-fold over the controls) was not impressive. With a similar intent, Andrade et al. [74] functionalized Pluronic^®^ F127 micelles with a CD44v6 Fab. In the complete formulation, the nanomicelles included niclosamide, a drug originally used as an anthelmintic and recently repurposed against cancer stem cells. The authors investigated the efficacy of their nanomedicines to target colorectal cancer stem cells and circulating tumor cells that express high levels of CD44v6. For this purpose, they sorted a colorectal cancer cell line according to CD44v6 expression (high versus low), thus confirming stemness features (including sensitivity to niclosamide) in CD44v6^high^ cells. Internalization of the targeted nanomicelles was significantly increased in CD44v6^high^ cells (>2.5-fold) versus CD44v6^low^. In vivo, the nanomicelles exhibited a good safety profile (up to 8-fold higher tolerable drug concentration compared with the free drug) and tumor accumulation in a xenograft model, although the increase due to CD44v6 targeting was not impressive (17% untargeted versus 22% targeted after 48 h circulation), suggesting that the EPR effect is likely dominant over antibody-mediated targeting. Although they had no effect on xenografted tumors, the CD44v6-targeted nanomicelles reduced the number of circulating tumor cells, showing potential anti-metastatic efficacy.

A way to simplify antibody coupling to nanoparticles—and avoid the complicated (and often poorly controlled) chemistry of crosslinking—consists of using bispecific antibodies that bind, from one side, the target molecule on cancer cells, and from the other, a component of the nanoparticle itself. Lin et al. [75] designed a bispecific antibody to simultaneously bind HER2 (scFv) and mPEG (Fab fragment). By simple incubation of the bispecific antibody with doxorubicin-embedding mPEG-liposomes (LipoDox^®^), the HER2 targeting component was loaded. The deriving immunoliposomes were capable of inducing apoptosis in ovarian cancer cells (5.4-fold compared with their untargeted counterpart) and to accumulate in models of ovarian cancer (220% compared with their untargeted counterpart). The same research group created a humanized bispecific antibody composed of a CD20 mAb (ofatumumab, approved for hematological malignancies) and a mPEG scFv to functionalize LipoDox^®^ by simple mixing [76]. This formulation was bound to a CD20-expressing lymphoblastoid cell line, where it was efficiently internalized (60% in 24 h) and showed 9-fold increased cytotoxicity compared with the untargeted liposomes. Coadministration of the bispecific antibody and LipoDox^®^ achieved a good control of tumor growth in a mouse model of B-cell lymphoproliferative disorder, leading to >90% of the mice cured and alive at end of the experiment (~100 days).

Trispecific antibodies have also been developed based on one anti-mPEG Fab and two scFvs against EGFR and fibroblast activation protein (FAP), respectively [77]. Micelles including mPEG, and stabilized with lecithin, were loaded with docetaxel and functionalized with the trispecific antibody or with one of the two bispecific combinations (mPEG + EGFR or mPEG + FAP). As preclinical models, the authors used cell lines overexpressing EGFR or FAP to mimic tumor and microenvironment cells, respectively, in different coculture ratios to validate the specificity of the different formulations. In vivo, all targeted formulations retarded tumor growth, although there was no additional benefit in the trispecific combination versus either bispecific combination.

Model nanoparticles that use antibody fragments as targeting moieties are reported in Table 5.

### 3.3. Active Targeting: Peptides as Targeting Moieties

A multitude of peptides have been identified that target cancer cells and/or tumor endothelial cells, which is also due to the implementation of in silico protein binding/3D docking simulations [78] and high-throughput peptide library screenings by phage display [79,80,81,82]. Some of these peptides mimic actual protein ligands, whereas others have been selected for their binding properties, independently from their primary sequence. A comprehensive examination of peptide-targeted nanoparticles in cancer therapy goes beyond the scope of the present review and has been excellently discussed elsewhere. Please refer to the reviews by Lu et al. and Seyyednia et al. [83,84] for a detailed and updated description of peptides that focus on tumor blood vessels and derived nanoparticles, respectively, and to the one by Sonju et al. [85] for peptide-functionalized liposomal formulations entrapping anticancer agents. Here, we report some recent examples.

#### 3.3.1. Arginine-glycine-aspartate (RGD) Peptides

The RGD motif binds to α_v_β_3_ and α_v_β_5_ integrins, which are overexpressed in angiogenic vascular endothelial cells as well as in some cancer cells, and for this reason it is a well-known tumor-targeting peptide that has been included in several formulations.

In some instances, RGD is produced with additional cysteines at each end to obtain a cyclized version by the formation of a disulfide bond (CRGDC, cRGD). Among the most recent publications, Wen et al. prepared biomimetic nanostructures, including a core of albumin–gefitinib (an EGFR tyrosine kinase inhibitor) and a coating of red blood cell membranes exposing the cRGD peptide [86]. This formulation was efficient for both in vitro and in vivo models of lung cancer, with no detectable systemic toxicity. In the study by Li et al. GNA002 (a recently developed inhibitor of enhancer of zeste homolog 2, EZH2) was loaded into nanocarriers, including cRGD (targeting portion), PEG-hydrazine (pH-sensitive portion), Arg_6_ (cell-penetrating peptide), and stearic acid [87]. The nanoparticles were designed to direct GNA002 to cancer cell nuclei and achieve the maximum effect on its pharmacological target (an enzyme involved in histone modification). cRGD was also used as a targeting moiety in prototypes of environmentally-friendly silk fibroin nanoparticles loaded with curcumin (an herbal supplement from *Curcuma longa*), demonstrating a specific uptake and significant impact on cancer cell metabolism in vitro [88]. Yuba et al. propose a combination of cRGD-mediated targeting and heat-induced doxorubicin release in a liposome coated by a thermosensitive polymer [89]. Another work describes nanoparticles based on DSPE-PEG-cRGD carrying a TQs-PEG4 photosensitizer for photodynamic therapy under 660 nm laser irradiation [90]. Han et al. report silver sulfide nanoparticles functionalized with cRGD for concomitant photothermal therapy and delivery of doxorubicin to models of triple negative breast cancer [91]. Hua et al. prepared gold-iron oxide nanoparticles modified with cRGD for fluorescence/MRI-based diagnostic imaging coupled with ROS production/cytotoxicity induced by X-ray irradiation in acidic conditions; this multimodal approach was successful in vivo, with tumor suppression in >80% of mice bearing a syngeneic xenograft model of breast cancer [92]. Zhang et al. synthesized gallic acid/Fe^2+^ nanoparticles carrying doxorubicin, including the cRGD-platinum(IV) prodrug [93]. These nanoparticles were capable of different functions, including chemotherapy, photothermal therapy, ROS-mediated cytotoxicity (apoptosis and ferroptosis), and diagnostic MRI. The authors propose applications in glioblastoma management, having demonstrated that their nanoparticles cross the blood–brain barrier, achieve tumor regression, and prolong survival in rodent models of intracranial tumors. A nanoparticle based on DSPE (core) and PEG-cRGD (shell), including calcium/phosphate ions absorbed into the shell, was designed to carry siponimod (BAF312, an inhibitor of sphingosine 1 phosphate receptor 1) [94]. This nanomedicine effectively decreased tumor growth and angiogenesis both in vitro and in breast cancer xenografts by impacting the signal transducer and activator of the transcription 3 (STAT3)/vascular endothelial growth factor (VEGF) signaling axis.

A variant of cRGD fused to a cell-penetrating peptide (Arg_8_-cRGD) has been included in a cholesterol-PEG-PEI nanoparticle for the delivery of a siRNA against Wee1, a nuclear kinase overexpressed in melanoma [95]. This formulation inhibited both primary tumor growth (by 85%) and secondary dissemination (by 66%) in a mouse model of metastatic melanoma. Another formulation for oligonucleotide delivery was based on cRGD-PEI/DSPE-PEG nanoparticles carrying an anti-survivin siRNA [96]. In vivo, tumor inhibition reached 74.7% in a model of liver cancer, with no systemic toxicity. Nanoparticles with different designs, but a similar intent, included a redox-responsive chitosan-based nanocarrier coated by albumin-cRGD, and were loaded with an anti-VEGF siRNA [97]. In addition to tumor-targeting, the dual-layer combination was conceived to provide a protective corona, and to avoid serum protein aggregation on the surface of chitosan nanoparticles.

In other instances, additional amino acids are fused to the RGD motif to improve cancer cell targeting and uptake. iRGD (CRGDK/RGPD/EC) is a variant created in Ruoslahti’s lab [98] by the insertion of a neuropilin-1–binding C-terminal rule (CendR) motif that promotes cell internalization. In vivo, the coadministration of iRGD and various chemotherapeutics (Abraxane^®^, doxorubicin, doxorubicin-encapsulating liposomes, or trastuzumab) enhanced both drug accumulation in tumor tissues and therapeutic efficacy. The authors demonstrated that iRGD increases the permeability of tumor blood vessels inducing the so-called bystander effect, with no need for chemical conjugation of the peptide to the drug/carrier, which has practical advantages [99]. The efficacy of co-administering paclitaxel-loaded PLGA nanoparticles and iRGD was recently confirmed in models of colorectal cancer [100]. Similarly, coadministration of iRGD and a sorafenib-loaded Fe-metal organic framework induced ferroptosis in a liver cancer cell line in vitro and inhibited tumor growth in vivo in a syngeneic model of mouse hepatoma [101]. Wan et al. prepared nanoparticles based on ε-poly-L-lysine bound to *cis*-aconitate and a thioketal group (ROS-sensitive) [102]. These nanoparticles were loaded with siRNAs against fibrinogen-like protein 1 (FGL1) and the checkpoint inhibitor programed cell death ligand 1 (PD-L1). Upon coadministration with iRGD, both siRNAs were efficiently delivered to cancer cells in vitro and they increased the numbers of tumor-associated immune cells in vivo, despite modest (albeit significant) inhibition of tumor growth in mouse models.

In further formulations, iRGD is embedded in the nanomedicine itself. For example, doxorubicin-loaded mesoporous silica nanoparticles coated with erythrocyte membranes and functionalized with iRGD efficiently reduced the growth of triple negative breast cancer models [103]. Moreover, doxorubicin-loaded tetrahedral framework nucleic acids decorated with iRGD demonstrated better antitumor and antiangiogenic activity than the free drug or untargeted formulations [104]. Mamnoon et al. prepared polymersomes by self-assembly of polylactic acid (PLA)-diazobenzene-PEG polymers including iRGD and carrying doxorubicin [105]. Drug release was responsive to hypoxia, as demonstrated in vitro (30% in normoxia, >95% in hypoxia), thus adding a further layer of specificity, in terms of delivery to the tumor microenvironment, in models of triple negative breast cancer. Ma et al. synthesized a thioketal-based (ROS-cleavable) dimer of ursolic acid (a terpenoid found in the peels of fruit and in some herbs with potential antitumor effects) and included this prodrug into iRGD-decorated DSPE-PEG nanoparticles; this nanosystem was validated in vivo in a model of gastric cancer [106]. Sui et al. manufactured an iRGD-functionalized nanoparticle to deliver a plasmid-encoded peptide inhibitor of chromosomal maintenance 1 (CRM1, a nuclear export factor involved in drug resistance) [107]. The inhibitor was expressed and induced regression of xenograft models of melanoma.

Along with chemotherapeutic drugs or oligonucleotides, iRGD targeted nanoformulations may include additional features. In one example, nanoparticles based on iRGD-DSPE-PEG, DPPC, and cholesterol were loaded with 10-hydroxycamptothecin and indocyanine green (a photosensitizer) and coated with liquid perfluoropentane. Upon ultrasonic irradiation, the latter undergoes liquid-gas transition thus disrupting the nanoparticle [108]; therefore, the nanomedicine accumulates at the tumor site (via EPR effect and iRGD targeting) where the therapeutic effect can be precisely triggered by release of both the chemotherapeutic drug and the photosensitizer, as demonstrated in vitro and in vivo. In a final example, Zhang et al. manufactured nanoparticles based on iRGD-DSPE-PEG, a doxorubicin prodrug, and poly(fluorene-covinylene), which induces ROS generation upon white light irradiation, thus allowing in situ activation and synergistic effects [109].

#### 3.3.2. Rationally Designed Peptides That Target Cancer Cells

Angiopep-2, a ligand of a low-density lipoprotein receptor-related protein (LRP1, overexpressed in gliomas and in cells of the blood-brain barrier), was functionalized on the surface of mesoporous silica nanoparticles loaded with paclitaxel [110]. These nanoparticles were more efficient than their untargeted counterpart, including a better tumor control and life extension in a rat model of orthotopic intracranial glioma. This same peptide was also linked on the surface of hyaluronic acid nanoparticles embedding gadolinium-diethylenetriamine pentaacetic acid (imaging agent for MRI) and irinotecan [111]. This formulation proved to increase irinotecan cytotoxicity in vitro.

A leptin-derived peptide (Lp31) was exposed on the surface of PEGylated liposomal doxorubicin (Caelyx^®^) [112]. Physiologically, leptin is an adipocyte-released hormone that decreases appetite; in cancer cells, leptin receptor Ob-R is often overexpressed and thus represents a potential target for drug delivery. The authors validated Lp31-Caelyx^®^ in the colon-26 mouse carcinoma model, documenting specific drug accumulation and inhibition of tumor growth, with a slightly better performance than untargeted Caelyx^®^.

dYNH, a ligand of EPH Receptor A2 (EphA2), was linked to the surface of multicomponent nanostructures (gold nanoparticle core, metal organic framework/mesoporous silica shell) carrying cisplatin and alpelisib (BYL71, an inhibitor of phosphoinositide 3-kinase, PI3K) [113]. Indocyanine green was also linked to the external layer to enhance the photothermic effect. The authors validated this multifunctional system in an orthotopic model of spinal metastasis from lung cancer, observing an enhanced uptake by cancer cells and therapeutic efficacy consistent with tumor regression and prevention of bone damage.

AE147, a ligand and antagonist of the urokinase plasminogen activator receptor (uPAR), was linked to PEG-liposomes carrying docetaxel [114]. The authors provide validation of specific binding to breast cancer cells (in vitro) and metastatic foci (in vivo).

A derivative of the decapeptide luteinizing hormone release hormone, in which Lys_6_ is a D-amino acid, (D-Lys)-(LHRH), was used to functionalize PEG-dendritic cholic acid nanomicelles to deliver paclitaxel with a redox-dependent release profile [115]. In vitro, uptake of these targeted nanomicelles in triple negative breast cancer cells was more efficient compared with the untargeted counterpart. In vivo, tumor accumulation was confirmed in three mouse models of breast cancer (cell line-derived xenograft, patient-derived xenograft, transgenic models). Superior therapeutic efficacy, compared with both the clinical formulation (Taxol) and the untargeted nanomicelles, was observed in a xenograft model.

A different approach was followed by Kim et al. who describe a short peptide substrate of cathepsin-B, of sequence FRRG, conjugated to doxorubicin and self-assembled in nanoparticles with the addition of Pluronic^®^ F68 [116]. In this case, targeting is achieved not via specific binding but via specific peptide cleavage by cathepsin-B, an enzyme overexpressed by cancer cells, with consequent disassembly of the nanoparticles and release of doxorubicin. Upon coadministration of intravenous nanoparticles and oral navitoclax (an inhibitor of the anti-apoptotic protein Bcl-2), a synergistic effect was observed in a xenograft model of drug-resistant breast cancer, with no side effects. Similarly, the cathepsin B-cleavable peptide GFLG was incorporated by Luo et al. in a branched polymer of the photosensitizer pyropheophorbide a [117]. Disulfide bonds were included to render the nanoparticles responsive to intracellular reductive conditions. This polymer self-assembled in regular nanoparticles, which were loaded with AZD2281, a poly(ADP)-ribose polymerase (PARP) inhibitor as an anticancer agent. High payloads of the active compounds were released intracellularly by the concomitant action of cathepsin B and GSH, inducing cell cytotoxicity and triggering photodynamic therapy. These nanoparticles were validated, with promising results, both in vitro and in a model of breast cancer.

#### 3.3.3. Affinity-Selected Peptides That Target Cancer Cells

Phage display-selected peptides are often being exploited as targeting moieties. Among these, a ligand of mitochondrial protein p32, of sequence CGKRK, was linked to DSPE-PEG and inserted into Caelyx^®^ at different peptide:liposome ratios (25, 50, 100, 200, 400). The best formulation was the one including 100 peptides per liposome, which was more efficient than Caelyx^®^ both in vitro and in vivo [118]. An EGFR-targeting peptide, GE11, was functionalized on the surface of liposomes carrying curcumin as a chemotherapeutic drug and indocyanine green as a photosensitizer [119]. In this system, NIR irradiation induces hyperthermia and triggers photothermal therapy. The authors demonstrated specific targeting to EGFR-expressing lung cancer cells in vitro, which, in concomitance with NIR irradiation, increases the release of curcumin, thus potentiating its effect. Another EGFR-specific peptide identified by phage display, QRH, was conjugated on the surface of polydopamine-based nanoparticles carrying doxorubicin and a phthalocyanine photosensitizer [120]. The structure of the dopamine polymer included thioketal bonds that are cleaved at the reductive intracellular conditions, leading to nanoparticle disassembly, drug/photosensitizer release, and cytotoxicity in vitro. In a xenograft model, treatment with the EGFR-targeted doxorubicin/photosensitizer-loaded nanoparticles, followed by NIR laser irradiation, led to an impressive result: complete regression of the tumor, at least for the duration of the experimental evaluation (14 days).

Peptide-directed tumor targeting is also possible without knowledge of the specific receptor. For example, SP94 (SFSIIHTPILPLGGC) was selected by phage display to bind the surface of hepatocellular carcinoma cells [121]. Doxorubicin-loaded polymersomes decorated with 30% SP94 were internalized by a cell line of hepatocellular carcinoma up to three times more than untargeted polymersomes, with specific cytotoxic effects that also outperformed LipoDox^®^ (non-targeted doxorubicin hydrochloride liposome formulation). Promising tumor accumulation in xenograft models (14.9% injected dose per gram; tumor-to-normal liver ratio ~6.9) and therapeutic effects in orthotopic models (survival: 76 days versus 45 days untargeted formulation and 33 days vehicle) were reported. Another targeting peptide, TMTP1 (NVVRQ), was isolated in a bacterial peptide display screening, and characterized as a ligand of highly metastatic cancer cells. A cyclic version of TMTP1 was linked to DSPE-PEG and co-assembled with lauric anhydride–cisplatin, cis-aconitic anhydride-PEG-paclitaxel, or both, to form nanoparticles of 159.2 ± 3.8 nm, 216.3 ± 4.7 nm, or 233.5 ± 8.6 nm, respectively [122]. The cancer-targeted, dual-loaded (carboplatin + paclitaxel) nanoparticles were highly cytotoxic in vitro, and accumulated in tumor tissues in vivo, with a peak at 24 h. In a xenograft model of squamous cell carcinoma of the uterus, these nanoparticles retarded tumor growth and prolonged survival. HN-1 peptide (TSPLNIHNGQKL), an additional motif isolated by phage display, was covalently linked to graphene oxide-PEG nanoparticles for the delivery of doxorubicin [123]. These nanoparticles targeted cancer cells both in vitro and in vivo in models of oral squamous cell carcinoma, and doxorubicin release was pH-responsive.

#### 3.3.4. Peptides That Target the Tumor Microenvironment

Several peptides have been designed to target the tumor microenvironment instead of, or in addition to, cancer cells themselves. LinTT-1, a phage-display-selected peptide ligand of the cell-surface receptor gC1qR, of sequence AKRGARSTA, was bound on liposomes coloaded with doxorubicin and sorafenib [124]. Consistent with the finding that gC1qR is overexpressed not only by breast cancer cells, but also by cancer-associated cells, these liposomes targeted M2-polaryzed macrophages with an uptake of 50%, thus potentially representing a microenvironment-directed nanomedicine. Another way to target M2 macrophages was applied by Parayath et al. [125], who used a phage display-derived peptide to functionalize hyaluronic acid-PEG nanoparticles self-assembled with miR125b (involved in several signaling pathways in cancer cells, including drug resistance and immune escape). The nanoparticles were tested in transgenic mouse models of KRAS-mutant pancreatic cancer, confirming specific accumulation at tumor sites, delivery of micro-RNA (miRNA) 125b, and conversion of macrophages from M2 (pro-tumor) to M1 (anti-tumor), compared with nanoparticles carrying a scrambled miRNA.

In addition to macrophages, angiogenic endothelial cells also play a crucial role in cancer progression/metastasis and represent an appealing therapeutic target. A peptide of sequence IELLQAR, isolated by phage display as a specific ligand of E-selectin (an adhesion molecule overexpressed by tumor-associated endothelial cells) was linked to PEG and SN38 (the active form of irinotecan). Upon self-assembly, this amphiphilic conjugate produced nanomicelles of hydrodynamic diameter ~60 nm that released the drug in acidic and reductive conditions [126]. With the combination of EPR effect and vasculature targeting, the nanomicelles accumulated at tumor sites in a xenograft model of colon cancer, reduced primary tumor growth, and prolonged survival with an effect similar to SN38. Moreover, they prevented lung colonization in a model of metastatic melanoma with an improved effect compared with SN38.

tLyP-1, a ligand of neuropilin-1 (overexpressed in tumor endothelial cells), was covalently linked to cholesterol and included in reconstituted lipoprotein nanoparticles coloaded with paclitaxel and GANT61 (an inhibitor of sonic hedgehog pathway) in the hydrophobic core [127]. The presence of apolipoprotein A-1 on the nanoparticle surface conferred additional tumor-targeting properties via binding to scavenger receptor B type I (overexpressed in tumor cells). Validation was achieved both in vitro and in vivo in models of metastatic triple negative breast cancer.

The described peptide-targeted nanoparticles are summarized in Table 6.

### 3.4. Active Targeting: Proteins as Targeting Moieties

Whole proteins can be exploited for the targeting of cells that overexpress the corresponding receptors. Among these, transferrin is a serum glycoprotein that transports iron in the bloodstream; ferritin, instead, is responsible for intracellular iron storage; lactoferrin carries iron in secretory fluids, such as milk, saliva, tears, and nasal secretions. All three proteins bind transferrin receptor 1 (TfR1), and lactoferrin also binds low density lipoproteins and asialoglycoprotein receptor which are often upregulated in cancer cells [128]. Several nanoparticles that use transferrin, lactoferrin, ferritin, or their derivatives as targeting moieties have been recently tested.

#### 3.4.1. Transferrin: Peptide Mimetics and Whole Protein

In order to optimize a transferrin receptor-directed nanoformulation, Mojarad-Jabali et al. prepared liposomes decorated with transferrin mimetic peptides, namely, T12, B6, and T7, and evaluated their capacity to cross the blood–brain barrier, distribute into the brain, and accumulate in gliomas [129]. From these preliminary experiments, T7 was chosen as the best performing peptide. T7-functionalized, vincristine-loaded liposomes were validated in vitro on glioma cells. As a further validation of this targeting system, the same transferrin mimetic was linked to the surface of PLGA-PEG-based nanoparticles loaded with seliciclib (a cyclin-dependent kinase, CDK inhibitor) [130]. The resultant transferrin receptor-targeted nanoparticles proved to selectively bind different cancer cells, with an uptake that was dependent on the levels of surface-exposed receptor.

Among specific applications, Zhu et al. [131] synthesized nanoparticles based on carboxymethyl chitosan/4-hydroxyphenylboronic acid pinacol ester and they were decorated with T7. The nanoparticles were loaded with docetaxel and curcumin, whose release was dependent on acidic pH and ROS, as evaluated in vitro in lung cancer cells. The T7-targeted, dual-loaded formulation outperformed all controls (included the free drugs), both in vitro and in vivo. The toxicity profile was promising, and tumor growth was suppressed (at least for the 11 days of experimental observation) in aggressive xenograft models of lung cancer.

Targeting of the transferrin receptor has also been achieved with full-length transferrin, as reported in several recent papers. Arumov et al. synthesized carbon-nitride dots that were covalently linked to both doxorubicin and transferrin [132]. The derived nanoparticles had an increased efficacy compared with the free drug (IC50 10–100 times lower, depending on the cell line). The formulation retarded tumor growth and prolonged survival in a patient-derived xenograft model of diffuse large B-cell lymphoma. Jin et al. used transferrin-targeted PEG-albumin nanoparticles to deliver doxorubicin in models of breast cancer, observing improved cancer cell cytotoxicity (in vitro) and inhibition of both primary and metastatic tumors (in vivo) compared with the free drug [133]. Li et al. used doxorubicin-loaded mesoporous silica nanoparticles with a coating of poly(N-isopropylacrylamide)-acrylic acid copolymer (pH-responsive portion) that included calcium phosphate and was functionalized with both transferrin and the RGD peptide [134]. The authors validated the efficacy of this formulation in vitro.

In addition, or in substitution to chemotherapeutic drugs, natural compounds with potential antitumor activity are often included in transferrin-targeted nanoparticles, with the aim of improving biodistribution and concentrating the active agent in likely therapeutic doses. Piperine, an alkaloid extracted from the fruits of *Piper nigrum* (white and black pepper), *Piper longum*, and *Piper officinarum*, was included in glycyrrhizic acid/PLGA nanoparticles decorated with transferrin [135], with a final size of 112.2 ± 1.27 nm and a negative charge. In vivo, transferrin-targeted nanoparticles did not outperform their untargeted counterpart, although they proved specific in different in vitro assays. Plumbagin, a naphthoquinone isolated from *Plumbago indica*, was loaded into phosphatidylcholine/DSPE-PEG nanoparticles functionalized with transferrin [136]. In a mouse model of melanoma, this formulation was capable of inducing tumor regression (10% of cases) and even disappearance (40% of cases). Erianin, a dibenzyl compound in *Dendrobium* extract, was embedded into transferrin-decorated liposomes with a final size of 88.3 ± 4.21 nm, and was validated in vitro and in vivo for their superiority over the free drug [137]. Thymoquinone, an essential oil found in the plant *Nigella sativa*, was included in three transferrin-decorated formulations: coloaded with gefitinib in mesoporous silica-PLGA (formulation NP-dual-1) or PEG-PLGA nanoparticles (NP-dual-2), or loaded in mesoporous silica nanoparticles covalently linked to gefitinib-loaded PEG-PLGA nanoparticles (NP-dual-3) [138]. Drug release was pH-dependent (>80% at pH 5.2) in all three formulations and led to the cell death of even gefitinib-resistant lung cancer cells; NP-dual-3 was the most effective, as evaluated in several in vitro assays on lung cancer cell lines. The superiority of NP-dual-3 over the other two formulations was also tested in vivo, in terms of toxicity, pharmacokinetics, and therapeutic effect. Interestingly, NP-dual-3 inhibited the growth of gefitinib-resistant subcutaneous lung cancer xenografts and prolonged survival up to 5 months; moreover, this formulation prevented lung metastases.

#### 3.4.2. Ferritin

The numerous applications of ferritin as a nanocarrier and targeting moiety have been treated in a recent review [139]. Here, we provide only a few updated examples. Dong et al. prepared paclitaxel-loaded nanocages of ferritin fused with a peptide inhibitor of extracellular-signal-regulated kinase (ERK), which is a cell transduction pathway often dysregulated in cancer [140]. The targeted nanocages were validated in vitro in 2D and in 3D (spheroids) cultures of breast cancer cells. Similarly, Huang et al. synthesized nanocages in which ferritin was fused to an α_2_β_1_-binding peptide of sequence DGEAGGDGEA [141]. These nanocages could load up to 458 molecules of doxorubicin and were capable of crossing the blood–brain barrier, as evaluated in vitro and in a mouse model of glioma where encouraging therapeutic effects were achieved upon intracranial administration (suppression of tumor growth and extended survival up to 80 days). As a final example, ferritin was modified by the inclusion of the tLyP-1 targeting/internalizing peptide and the derived nanocages were loaded with paclitaxel [142]. Specific tumor cell accumulation and cytotoxicity were confirmed both in vitro and in vivo in models of breast cancer.

#### 3.4.3. Lactoferrin

Lactoferrin is also emerging as a promising targeting moiety for various types of cancer, due to its binding to different receptors. Abdelmoneem et al. conjugated docetaxel and celastrol (an inhibitor of nuclear factor kappa-light-chain-enhancer of activated B cells, NF-kB) to lactoferrin [143]. This conjugate self-assembled in nanoparticles of 157.8 nm that proved more efficient than the free drugs in inhibiting tumor growth and extending lifespan in a model of breast cancer. Similarly, Narayana et al. loaded carboplatin and etoposide onto lactoferrin nanoparticles [144]. By setting up a novel synthesis protocol, Janjua et al. produced ultrasmall (~30 nm) silica nanoparticles with large pores (>7 nm), loaded with doxorubicin and functionalized with lactoferrin [145]. In different in vitro assays, these nanoparticles were capable of crossing the blood–brain barrier (a feature that free doxorubicin does not possess), were internalized by glioma cells, potentiated doxorubicin-induced apoptosis, and penetrated in glioma cell spheroids more than their untargeted counterparts. Nisha et al. synthesized PEGylated liquid crystalline nanoparticles decorated with lactoferrin and loaded with imatinib (a broad-spectrum tyrosine kinase inhibitor) [146]. These cubical nanoparticles had a size of 120.40 ± 2.75 nm and a positive charge. Upon oral administration, they showed better pharmacokinetics (sustained drug levels at tumor sites up to 12 h) and biodistribution (~2.5-fold higher tumor accumulation compared to the free drug) in rats with carcinogen-induced liver cancer, which was paralleled by their increased antitumor efficacy. Zhang et al. synthesized doxorubicin-embedding nanogels functionalized with lactoferrin and phenylboronic acid for dual targeting of glioma [147]. Doxorubicin release was regulated by redox conditions due to the presence of disulfide bonds in the structure. In vitro, cell uptake was superior for the dual-targeted formulation compared with each single-targeted and doxorubicin-nanogel. In experimental rats, the dual-targeted formulation outperformed the others in terms of both pharmacokinetics (increased area under the curve) and brain accumulation (4.67-fold higher than the formulation targeted via phenylboronic acid only).

#### 3.4.4. Folic Acid (Folate)

Folate is a widely exploited targeting moiety, due to the overexpression of its receptor on the surface of cancer cells. Examples include the functionalization of superparamagnetic iron oxide nanoparticles at sizes of 178.1 ± 3.12 nm loaded with paclitaxel [148], human serum albumin nanoparticles at sizes of 180 ± 12.31 nm coloaded with paclitaxel and 2-methoxyestradiol (chemosensitizer) [149], and PEG-paclitaxel nanocrystals at sizes of 201.90 ± 2.92 nm [150], all with improved cell uptake and tumor accumulation, as well as better pharmacokinetics and therapeutic profiles in rat and mouse models, compared with the free drug (Taxol).

Other examples involve nanoparticles loaded with more than one drug (including natural/traditional medicine compounds) and/or with more than one application. Karpuz et al. produced liposomes coloaded with paclitaxel and vinorelbine, which were also radiolabeled with tecnetium-99 metastable to include a diagnostic function [151]. The targeted liposomes were taken up by tumor tissues in the mouse model of Lewis lung carcinoma, with positive outcomes (delayed tumor growth and metastatic dissemination, lesser toxicity compared with the free drug). Lin et al. prepared nanoparticles based on doxorubicin and berberine (a benzylisoquinoline alkaloid found in plants of the genus *Berberis*) embedded in a DSPE-PEG-folic acid shell and exposing hyaluronic acid for additional tumor targeting [152]. The structure was sensitive to pH and was degraded into the lysosomes, exposing the internal, positively charged core and allowing lysosomal escape, drug delivery to the mitochondria, and consequent onset of apoptosis. Similarly, Guo et al. synthesized nanoparticles with the self-assembly of two polymers, namely, (oligo)hyaluronic acid-mannose-folic acid and astragalus polysaccharide-dithiodipropionic acid-paeoniflorol [153]. The nanoparticles were loaded with both paclitaxel and baicalein (a flavone isolated from the roots of *Scutellaria baicalensis* and *Scutellaria lateriflora*). The latter is an interesting pharmaceutical compound in that it has been shown to interact with tumor-associated macrophages and induce a change in their polarity from M2 to M1. The dual loaded, tumor-targeted nanoparticles proved more efficient in suppressing tumor growth, with lower paclitaxel-related side effects, compared with their untargeted counterpart and to the free drug. Mousazadeh et al. produced PEI-β-cyclodextrin nanoparticles decorated with folic acid for the codelivery of doxorubicin and a siRNA against human telomerase reverse transcriptase (hTERT) [154]. Folic acid was bound via a redox-responsive disulfide bond, siRNA oligonucleotides were associated by electrostatic interaction, and doxorubicin release was dependent on pH. The authors validated specific targeting, internalization, siRNA transduction, and cytotoxicity to breast cancer cells in vitro.

Model nanoparticles that use proteins as targeting moieties are reported in Table 7.

### 3.5. Active Targeting: Aptamers as Targeting Moieties

Aptamers are small, non-immunogenic, high-affinity nucleic acid ligands that bind proteins [155]. Chemically, aptamers are G-rich oligonucleotides that are linked to form quadruplex structures. They were initially isolated in the early 1990s by the SELEX technique (systematic evolution of ligands by exponential enrichment) [156], which has been further improved to identify targeting moieties for cell surface molecules that would allow receptor-mediated internalization [157,158]. An example of an aptamer identified by SELEX is described by Go et al. [159]. The authors synthesized 20 nm gold nanoparticles loaded with doxorubicin and linked them via a disulfide bond to an aptamer ligand of prion protein C (PrP^C^), a cell surface glycoprotein overexpressed in cancer. These nanoparticles were more efficient than their untargeted counterpart in inducing apoptosis of colon cancer cells in vitro.

#### 3.5.1. Aptamers That Target the Prostate-Specific Membrane Antigen (PSMA)

The first report of an aptamer-bound nanoparticle for targeted delivery was published in 2004 [160]. The authors synthesized PLA-PEG nanoparticles that included rhodamine-labeled dextran and were decorated with a PSMA-targeting RNA aptamer (A10). These conjugates were able to specifically bind to, and be internalized by, PSMA-positive prostate cancer cells, unlike their untargeted counterpart. Recently, a PSMA-binding aptamer was used for the targeted delivery of paclitaxel and siRNAs to prostate cancer models [161]. In this study, paclitaxel was included in the hydrophobic core of self-assembled DSPE-PEG-calcium phosphate nanoparticles functionalized with the aptamer. Different siRNAs (against the androgen receptor, β-tubulin, and C-X-C chemokine receptor type 4, CXCR4) were also absorbed in the final formulation, giving nanoparticles at sizes of 119.20 ± 1.34 nm a negative charge. Both paclitaxel and the siRNAs were released faster at pH 5.5 compared with pH 7.4. Nanoparticles including both paclitaxel and the siRNA had superior antitumor effects, compared with nanoparticles loaded with single agents, as evaluated in vitro (inhibition of cell migration) and in vivo (delayed tumor growth and inhibition of metastasis in xenograft and orthotopic models, respectively), as well as in paclitaxel-resistant prostate cancer models. In the described experiments, the aptamer-targeted nanoparticles were more efficient than their untargeted version.

#### 3.5.2. Aptamers That Target Nucleolin

An aptamer extensively exploited as cancer-targeting moiety is AS1411, a specific ligand of nucleolin, overexpressed on the surface of cancer cells and angiogenic/tumoral endothelial cells [162]. In a recent application, AS1411 was used to decorate mesoporous polydopamine nanoparticles for docetaxel delivery to prostate cancer cells; these nanoparticles allowed a combined photothermal therapy upon irradiation with NIR light [163].

Doxorubicin is often utilized in AS1411-targeted nanoparticles, either as single agent or in combination. In a work by Zhuang et al. [164], mesoporous silica nanoparticles were loaded with doxorubicin and decorated with AS1411 and a siRNA against the tyrosine kinase receptor 2 (Tie2, overexpressed in tumor endothelial cells and macrophages), which were both linked via redox-responsive disulfide bonds. Targeting through AS1411 increased cell uptake and improved doxorubicin efficacy, as the authors demonstrated in both cultured breast cancer cells and in a mouse model of metastatic breast cancer.

AS1411-targeted mesoporous silica nanoparticles for the codelivery of doxorubicin and anti-miR21 (a miRNA with oncogenic properties) have been recently reported [165]. The nanoparticles were coated with chitosan, which provided electrostatic attachment of both AS1411 and anti-miR21. The cumulative release of doxorubicin was higher in acidic environments (64.28% within 6 h at pH 5.5) compared with physiological conditions (67.39% within 48 h at pH 7.4). Treatment with these nanoparticles resulted in enhanced cytotoxicity in vitro and improved survival of mice xenografted with a syngeneic model of colon cancer (all alive at day 21), despite the reported non-impressive impact on tumor growth inhibition.

In a work by Sathiyaseelan et al. doxorubicin and 5-FU were coloaded into gold nanocarriers coated with chitosan and surface-functionalized with AS1411, giving nanoparticles at sizes of 196.2 ± 2.89 nm with a positive charge [166]. Drug release was dependent on pH and specific uptake was confirmed in vitro on glioblastoma cells.

In another study, 10 nm gold nanoparticles were coated with a scaffold of polyacrylic acid, five structural and functional DNAs (including AS1411), a VEGF-directed siRNA, and doxorubicin [167]. The derived gold/DNA nanocages were held together by both DNA complementarity and covalent binding through a peptide substrate of MMP-2. The nanocages were evaluated in vitro (cellular uptake and intracellular distribution) and in vivo (tumor accumulation and therapeutic effect in an orthotopic mouse model of lung cancer). Drug and siRNA release were obtained via peptide cleavage by MMP2; in addition, photothermal treatment was achieved by laser NIR irradiation of the tumor mass, resulting in an almost complete tumor regression after 36 days of treatment, with 60% of the animals still alive at the end of the experiment (day 125), compared with <35% of those treated with VEGF-siRNA+AS1411 nanocages not loaded with doxorubicin. The outcome for the other treatment arms was dismal: no animal survived at day 30 (VEGF-siRNA only) or 90 (nanocages with VEGF-siRNA only).

Chen et al. described polyamidoamine (PAMAM) dendrimer nanoparticles embedded with a superparamagnetic zinc-doped iron oxide octahedral core and surface-modified with PEG and fluorescent dyes [168]. These nanoparticles were loaded with doxorubicin and a siRNA against heat shock protein 90/70 (HSP70/HSP90) to increase sensitivity towards chemotherapy- and hyperthermia-induced cytotoxicity. The inclusion of AS1411 as targeting moiety increased the specific uptake and tumor accumulation of the nanoparticles in vitro and in vivo.

In other prototypes, the main encapsulated drug was camptothecin. After testing camptothecin-loaded polymersomes with different molar ratios of PEG-PLA:DPPC, Zahiri et al. [169] chose the formulation 75:25 as being superior in terms of stability and drug loading. The PEG-PLA:DPPC polymersomes were functionalized with AS1411 and tested on colon cancer cell lines, showing specific uptake/cytotoxicity in vitro and tumor accumulation in vivo. In another work, PLA-PEI-based nanomicelles were designed for the codelivery of camptothecin (included in the hydrophobic core) and an anti-survivin short hairpin RNA (shRNA) (electrostatically linked to the positively charged shell) [170]. The resulting nanomicelles were further covered with poly(carboxylic acid) dextran, and AS1411 was covalently bound to their surface. In vitro assays demonstrated specific uptake by receptor-mediated endocytosis, increased drug accumulation, and induction of apoptosis in colon cancer cell models. In vivo, both the untargeted and the AS1411-targeted nanomicelles inhibited tumor growth with rates of 87% and 93%, respectively.

A nature-friendly application of AS1411 nanocarriers included starch nanoparticles for the delivery of *p*-coumaric acid (a derivative of plant extracted cinnamic acid with cytostatic properties on cancer cells) to cell models with triple-negative breast cancer [171]. These nanoparticles had a size of 218.97 ± 3.07 nm and a negative charge, with a good encapsulation efficiency (~80%), and a rapid release over 5 h in acidic conditions (pH 5.4). The presence of AS1411 increased specificity and cytotoxicity/apoptosis in vitro.

Finally, innovative technologies exploit AS1411-directed targeting in combination with topical gels. For example, Rata et al. prepared nanocapsules loaded with 5-FU and decorated with AS1411, to be included into a sodium alginate/hyaluronic acid gel for topical treatment of skin cancer [172]. This formulation was a non-irritant, as evaluated in several in vitro and in vivo toxicity (hemolysis, irritation, cytotoxicity) and permeation/diffusion tests. Similarly, AS1411-targeted gold nanoparticles loaded with an acridine orange derivative (C8) or imiquimod (an immunomodulator approved to treat cutaneous malignancies) were incorporated in a PEG-based gel for topical treatments in the female genital tract [173]. The nanoparticles were characterized in vitro, and permeation of the gel formulation was evaluated in Franz cells using porcine vaginal tissue. Neither study, however, evaluated the therapeutic potential of these gel formulations in vivo.

#### 3.5.3. Aptamers That Target Mucin 1

Another aptamer that has been included as a targeting moiety for drug delivery is a ligand of mucin 1, a protein under-glycosylated and overexpressed in several types of cancer. Bagheri et al. [174] designed mesoporous silica nanoparticles loaded with doxorubicin and functionalized with a mucin 1-binding aptamer (covalently bound) and two adenosine triphosphate (ATP)-binding aptamers (associated via partial sequence complementarity). In the highly metabolic tumor microenvironment, where ATP concentration is ~10 mM, the ATP-binding aptamers were demonstrated to favor cell uptake and release of the drug payload. On the contrary, at the low concentration of ATP in the circulation (1 mM), the nanoparticle was stable and doxorubicin leakage was negligible. In vitro testing confirmed binding and entry specificity in mucin 1-expressing cancer cells compared with mucin 1-negative cells. The presence of the ATP-binding aptamers conferred a higher cytotoxic effect (in vitro) and antitumor efficacy (in vivo) compared with the free drug and with nanoparticles functionalized with scrambled aptamers.

In another study, doxorubicin-loaded superparamagnetic iron oxide particles conjugated to the fluorescent siderophore pyoverdine and linked to the mucin 1-binding aptamer were synthesized [175]. The derived nanoparticles had a size of 127.6 ± 5.8 nm and a negative charge, and they released the drug preferentially in acidic conditions (pH 5.5). In vitro validation confirmed the specific uptake and cytotoxicity in mucin 1-expressing cells compared with mucin 1-negative cells. In vivo testing showed delayed tumor growth (with volumes 2–3 times smaller than the controls at day 20) and prolonged survival (100% animals alive at day 20) compared with the free drug and to an untargeted formulation in a mouse model of colon cancer. The superparamagnetic iron oxide core of the nanoparticles was also exploited as an imaging agent for MRI.

Concomitant therapeutic and diagnostic attributes have been included in other nanoparticles based on hydrogel/quantum dots for fluorescent imaging [176]. These nanoparticles were functionalized with the mucin 1-targeting aptamer and coloaded with paclitaxel and sodium oxamate (an inhibitor of lactate dehydrogenase, an enzyme highly active in cancer cells with glycolytic metabolism). The specificity and efficacy of these nanoparticles was evaluated in vitro in a cell line of breast carcinoma.

A final example of the mucin 1-targeted aptamer is reported by Nabi et al. [177]. PAMAM polymers were linked to diethylenetriaminepentaacetic acid and PEG, loaded with gefitinib and functionalized with the aptamer. The deriving nanoparticles were also labeled with the radiotracer gallium-67. Gefitinib was slowly released over 7 days; the targeted nanoparticles were cytotoxic to mucin 1-expressing cancer cells in vitro and they accumulated at tumor sites in vivo, as revealed by nuclear medicine imaging.

#### 3.5.4. Miscellaneous Aptamers That Target Cancer Cell-Surface Proteins

Epithelial cell adhesion molecules (EpCAM) CD133, EGFR, and HER2 are additional targets overexpressed in cancer cells, cancer stem cells, and/or tumor endothelial cells, which have been exploited in the design of aptamer-functionalized nanoparticles. Here, we provide some very recent examples.

Doxorubicin-loaded PEGylated liposomes were decorated with an EpCAM-binding aptamer [178]. In vitro, these liposomes were specifically incorporated by cancer cells and induced cytotoxicity. In vivo, they accumulated into tumor masses in a mouse model of colon cancer, where immunostaining of tumor-extracted cells revealed an overexpression of EpCAM compared with cultured cells. In another study [179], paclitaxel-loaded superparamagnetic iron oxide nanoparticles, functionalized with the EpCAM aptamer, were internalized in higher amounts and resulted in a higher cytotoxicity than their untargeted version on a panel of cell lines in vitro.

Smiley et al. [180] describe poly(styrene-b-ethylene oxide)-PLGA polymeric nanomicelles coloaded with temozolomide and idasanutlin (RG7388, an inhibitor of mouse double minute 2, MDM2) and decorated with an aptamer for CD133, which is a surface glycoprotein overexpressed by cancer stem cells in glioblastoma multiforme. The deriving nanoparticles were also labeled with a radiotracer, zirconium-89, for imaging analysis. The authors provide a thorough characterization of this formulation, along with in vitro experiments demonstrating efficient targeting and killing of glioblastoma cancer stem cells. Similarly, Behrooz et al. [181] included temozolomide and paclitaxel into PAMAM-based dendrimer nanoparticles functionalized with B19, a selective aptamer ligand of CD133. Again, the targeted formulation exhibited specific antitumor effects in vitro (downregulation of genes involved in autophagy and drug resistance, inhibition of cell proliferation) on a glioblastoma multiforme cell line.

Zhu et al. [182] synthesized PAMAM and fluorinated PAMAM dendrimers linked to an EGFR-targeting aptamer, where the fluorinated dendrimer had the function of oxygen carrier. The nanoparticles were coloaded with gefitinib and hematoporphyrin (a photosensitizer for photodynamic therapy), which were efficiently released in acidic pH conditions. Treatment of EGFR-expressing non-small cell lung cancer cells with aptamer-targeted, dual loaded nanoparticles led to specific binding and uptake, inhibition of cell proliferation, and induction of apoptosis. In addition, hypoxic conditions were reversed by the dendrimer-carried oxygen. Laser irradiation at 630 nm led to an increase in intracellular ROS due to hematoporphyrin. Importantly, the synergistic effects were also active on gefitinib-resistant cancer cells in vitro. Another EGFR aptamer-targeted approach is described by Agnello et al. [183]. The authors prepared PLGA-PEG-based nanoparticles which were covalently linked to the CL4 (EGFR-specific) aptamer, including cisplatin plus a dye (4,4-difluoro-1,3,5,7-tetramethyl-4-bora-3a,4a-diaza-s-indacene, BODIPY^TM^ 505/515, or Cy7), for fluorescent tracing. Aptamer-targeted nanoparticles with different combinations of single and double agents were validated for their specificity and cytotoxicity in vitro on cell lines of triple negative breast cancer, using EGFR negative cells and nanoparticles functionalized with a scrambled aptamer as controls. In vivo, mice xenografted with breast cancer cells were administered fluorescent cisplatin-loaded nanoparticles. Tumor targeting was confirmed by fluorescence reflectance imaging. Although tumors continued to expand in all other control arms (vehicle, free cisplatin, and cisplatin-nanoparticles decorated with scrambled aptamers), tumor growth was controlled by CL4-functionalized nanoparticles at least up to the end of the experiment (day 24).

Finally, mesoporous silica nanoparticles coated with chitosan (as a pH-responsive moiety) and functionalized with an HER2-specific aptamer, were loaded with doxorubicin and successfully validated in models of triple-negative, HER2-expressing breast cancer cells [184]. The nanoparticles described in this paragraph are summarized in Table 8.

### 3.6. Active Targeting: Sugars as Targeting Moieties

Cell surface and extracellular sugars are well-known recognition molecules for cell–cell and cell–matrix interactions. Due to this feature, and since some types of saccharides are specifically overexpressed in tumor tissues, they have been employed as targeting moieties in the design of cancer nanotherapeutics.

#### 3.6.1. Hyaluronic Acid and Chondroitin Sulfate

Hyaluronic acid is a polymer of D-glucuronic acid and N-acetyl-D-glucosamine, where the number of repetitions is variable and may add up to 25,000. This sugar binds to different receptors, namely, the cluster of differentiation 44 (CD44), the lymphatic vessel endocytic receptor (LYVE-1), and the receptor for hyaluronic acid-mediated motility (RHAMM) [185,186,187]. All these receptors are upregulated in tumor cells and have an active role in cancer progression.

In a very recent work, PLGA-based nanoparticles were functionalized with hyaluronic acid of different weights (200, 800, and 1437 kDa) and their targeting properties were evaluated on CD44-overexpressing cancer cells [188]. The authors observed the highest selectivity and uptake by cancer cells, compared with normal fibroblasts, for nanoparticles decorated with the 800 kDa version of hyaluronic acid. Du et al. synthesized liposomes decorated with a much smaller version of hyaluronic acid, namely, a tetramer with molecular weight of 776.7 Da, to deliver oxaliplatin [189]. These liposomes induced apoptosis and necrosis in tumor cells in vitro and exerted antitumor effects in a xenograft model of colon carcinoma by increasing the inflammatory/immune cell infiltrate in tumor masses.

Several types of hyaluronic acid-decorated nanoparticles are now being engineered, most of which also include pH/redox-sensitive moieties, for the encapsulation and controlled delivery of one or more drugs. Xia et al. prepared chitosan-based nanoparticles, where hyaluronic acid was linked via a pH-responsive disulfide bond [190]. The nanoparticles self-assembled via electrostatic interactions and were successively stabilized by crosslinking. Depending on the chitosan/hyaluronic acid ration, they either had a positive (ratio = 0.1) or negative (ratio = 0.5) charge, sizes in the ranges 337.1–582.2 nm or 263–274.2 nm, and doxorubicin loading efficiency of 45.7% or 14.2%, respectively. At pH 4.5, and in reductive conditions, the drug was released up to ~88%, as investigated in vitro in a human breast cancer cell line. In another work, hyaluronic acid-decorated chitosan nanoparticles, at sizes of 180 ± 8.3 nm and with a positive charge, were coloaded with doxorubicin and miR-3a [191]. The nanoparticles exhibited a slow-release rate over 96 h, efficiently delivering their therapeutic payload to breast cancer cells in vitro, and inducing a ~74% inhibition of tumor growth in a breast cancer xenograft model.

Curcio et al. included doxorubicin into hyaluronic acid-albumin nanoparticles that self-assembled with an average size of 70 nm [192]. The stability of these small albumin particles was influenced by redox conditions, so that the drug could be released in a reductive environment. Specific uptake and cytotoxic effects were confirmed in vitro in a breast cancer cell line compared with nontumoral cells. In another study, 176 nm negatively charged albumin nanoparticles, including a lipid (D-α-tocopherol acid PEG-succinate, TPGS) and a CD44-binding portion (the polysaccharide chondroitin sulfate, structurally similar to hyaluronic acid), were loaded with paclitaxel [193]. In vitro, these nanoparticles entered breast cancer cells via CD44-mediated endocytosis and delivered high amounts of paclitaxel intracellularly. In vivo, they demonstrated a prolonged circulation time and specific tumor accumulation at 24 h post injection, with beneficial effects on tumor growth (inhibited by ~75%) and survival (100% of mice alive at 32 days versus none in the vehicle arm and 20% in the free drug arm) in a mouse xenograft model of breast cancer.

Hu et al. prepared PEI-PLGA-hyaluronic acid nanoparticles loaded with olaparib (a poly(ADP-ribose) polymerase, PARP, inhibitor) [194], having a hydrodynamic diameter of ~160 nm and a negative charge. These nanoparticles had efficient drug loading rates (>75%) and showed antitumor activity both in vitro (induction of apoptosis in breast cancer cells) and in vivo (inhibition of xenografted tumor growth).

In another example of application, dasatinib was conjugated to hyaluronic acid by a pH-sensitive bond, followed by inclusion in TPGS copolymers [195]. The derived nanoparticles had a hydrodynamic diameter of 82.23 ± 1.07 with (i) enhanced drug release in acidic conditions (pH 5.5) compared with physiological conditions (pH 7.4); (ii) increased uptake and cytotoxicity compared with their untargeted counterpart, also found in cisplatin-resistant cells; and (iii) no apparent cytotoxic effects in mice compared with the free drug, which caused damage to liver and kidney.

In a further prototype, cantharidin (a natural compound derived from *Mylabris cichorii*) was loaded into hyaluronic acid-mPEG copolymer-based nanoparticles of 119.3 nm [196]. The pharmacokinetics of this formulation was improved compared with the free drug, and its clearance rate was 0.41 times lower. In vivo imaging by fluorescence endomicroscopy and optical imaging revealed specific accumulation (compared with the untargeted nanoparticles) in a xenograft model of hepatocellular carcinoma, with inhibition of tumor growth at ~66% and extended survival.

Among the most recent examples of dual drug delivery, cabazitaxel was conjugated to hyaluronic acid, and the derived prodrug was coloaded with orlistat (an anti-obesity drug suggested to be repurposed to pancreatic cancer) in a PLGA-based nanocarrier [197]. The derived nanoparticles had a size of 150.9 nm and included cabazitaxel:orlistat in a 1:2 ratio, which was found to be optimal for synergistic effects, as confirmed in vitro and in vivo in prostate cancer models.

An interesting application in terms of biocompatibility involves the use of regenerated silk fibroin as the main component of hyaluronic acid-modified nanoparticles for the delivery of curcumin and 5-FU [198]. These nanoparticles had a size of ~200 nm and a negative charge, and they responded to environmental stimuli such as low pH, reductive conditions, ROS, and lysosomal hyaluronidase by releasing higher amounts of the drugs. The optimal ratio between curcumin and 5-FU was set at 1:1.2 to achieve the best synergistic effect. The authors demonstrated a superior performance of hyaluronic acid–nanoparticles compared with non-functionalized nanoparticles both in vitro (induction of apoptosis, inhibition of cell migration) and in vivo (delayed tumor growth in a xenograft model of breast cancer). Similarly, Al-Jubori et al. [199] engineered Poloxamer407-based nanoparticles, including both resveratrol (a phenol derived from the skin of grapes, berries, and peanuts) and tamoxifen (an anti-estrogen used as treatment for hormone-responsive breast cancer). The nanoparticles were covered by alternate layers of chitosan (positive charge) and hyaluronic acid (negative charge), and they were validated for their capacity to kill breast cancer cell lines in vitro. A toxicity analysis in vivo showed no damage to several organs (heart, lung, liver, spleen, and kidney).

Finally, three therapeutic agents with different chemical properties, namely, doxorubicin, camptothecin, and an aptamer inhibitor of Forkhead Box M1 (FOXM1), have been included in hyaluronic acid-decorated polymersomes [200]. Doxorubicin was loaded into the internal aqueous core, camptothecin was included in the lipidic shell, and the aptamer was conjugated to hyaluronic acid. This formulation was particularly efficient in terms of controlled release over 200 h, synergistic cytotoxic effect, induction of apoptosis in lung cancer cells in vitro, and tumor accumulation in vivo after 24 h in a xenograft model of lung cancer.

#### 3.6.2. Fucoidan

Another sugar that has been very recently used for nanoparticle functionalization is fucoidan, a sulfated polysaccharide extracted from brown algae which targets P-selectin, a protein overexpressed by tumor endothelial cells. In a study by Bala Tannan et al. S63845 (an inhibitor of myeloid cell leukemia 1, MCL1) and venetoclax (an inhibitor of B-cell lymphoma 2, Bcl-2) were included in fucoidan-based nanoparticles [201]. In a mouse model of diffuse large B-cell lymphoma, nanoparticle codelivery of S63845 and venetoclax resulted in accumulation at the tumor site, as evaluated by in vivo fluorescent imaging with an IVIS instrument. Mass spectrometry analysis of venetoclax concentrations showed an increase of 27% in the tumor and a decrease of 43% in the plasma compared with systemic administration of the free drug. Codelivery of S63845 and venetoclax in the fucoidan nanoparticles led to remission of experimental tumors in mice.

In two companion studies, DuRoss et al. evaluated nanoparticle-mediated codelivery of talazoparib (another PARP inhibitor) and temozolomide [202,203]. Nanoscale metal organic frameworks were prepared from hafnium plus 1,4-dicarboxybenzene, coated with fucoidan and loaded with both drugs, to obtain nanoparticles with radiation-stimulated delivery properties for chemoradiation strategies, which also exploited the overexpression of P-selectin during radiotherapy of cancer. The papers include nanoparticle characterization, in vitro toxicity studies on a colorectal cancer cell line, and in vivo tumor accumulation/antitumor efficacy studies. Both drugs were efficiently encapsulated (90% temozolomide, 54% talazoparib) and released, ~40% in the first 24 h and then up to 60% (temozolomide) or 100% (talazoparib) over 7 days. Based on these data, the authors performed preliminary studies with different administration schedules and chose the most aggressive non-toxic regimen (twice daily, for 5 consecutive days) for the in vivo trials. In mice that received this treatment in concomitance with radiation therapy (2 Gy, 2 sessions), tumor growth was delayed, and lifespans were prolonged.

From the perspective of natural compound-based nanoparticles, an attention-grabbing prototype has been manufactured by Zhang et al. [204] using ginger lipids to build plant-derived edible nanoparticles coated by a layer of ε-poly-lysine (positive charge) and a layer of fucoidan (negative charge). Doxorubicin was loaded as a model chemotherapy, and the efficacy of these nanoparticles was characterized in vitro and in vivo. Biodistribution studies revealed a 4.4-fold accumulation in tumor vasculature compared with untargeted nanoparticles, leading to a significant reduction of tumor growth, and an excellent biocompatibility of this nature-friendly formulation.

The nanoparticles described in this paragraph are summarized in Table 9.

**Figure 2:**
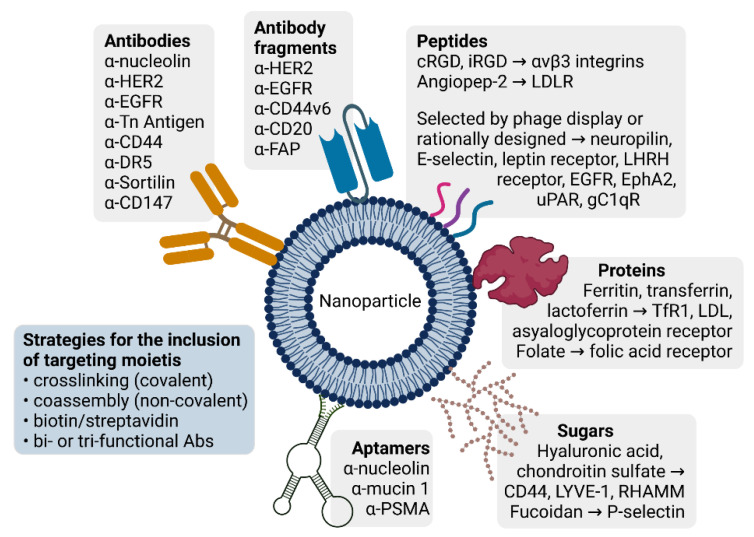
Examples of targeting moieties and their corresponding cell-surface targets that have been exploited for the specific delivery of chemotherapy to cancer cells. A liposome is reproduced in this illustration as a model nanocarrier. A schematic summary of general strategies utilized to couple the targeting moieties to a carrier nanoparticle is also included. Created with BioRender.com.

To conclude this section, the described systems for active targeting of nanoparticles at tumor sites are schematized and explained in Figure 2, with reference to both the targeting moiety and the cognate target on the surface of cancer cells, cancer stem cells, tumor-associated macrophages, or tumor endothelial cells. As an integration of the examples provided here, a broad discussion of approaches specifically targeting the tumor microenvironment is provided in the excellent review by Li and Burgess [205].

## 4. Multidrug Resistance

A limit of chemotherapy is the initial (primary) or treatment-induced (acquired) unresponsiveness to one or more drugs. To further complicate the situation, upon cancer progression or recurrence, multidrug resistance is often established, meaning that the tumor becomes unresponsive to both similar and structurally-unrelated classes of cytotoxic agents. Resistance involves biochemical and pharmacological mechanisms, including decreased uptake, increased efflux, inactivation, inability to convert the drug into its active form, upregulation of DNA repair systems, and alteration of intracellular pathways. Plasma membrane transporters of the ATP-binding cassette (ABC) family, such as P-glycoprotein (P-gp, also called multidrug resistance protein 1, MDR1), are responsible for increasing drug efflux and are often overexpressed in advanced tumor stages [206]. A first strategy to circumvent multidrug resistance is to administrate an inhibitor of an efflux pump along with the drug. A second strategy involves the coencapsulation of drugs with a different structure and/or activity to obtain a combined effect on different intracellular pathways. For example, paclitaxel, doxorubicin, or gemcitabine have been coformulated with (i) other chemotherapeutic (or repurposed) drugs, (ii) natural compounds, (iii) small-molecule inhibitors, and/or (iv) nucleic acids (e.g., siRNAs and miRNAs).

### 4.1. Reverting Multidrug Resistance by Nanoparticle-Mediated Gene Silencing or Chemical Inhibition of Efflux Pumps

Co-administration of chemotherapeutics and inhibitors of the efflux pumps has proven to be poor in terms of effectiveness when the compounds are administered separately. Instead, including them in a nanoparticle is advantageous, as demonstrated by the pioneering work of Meng et al. [207]. They prepared mesoporous silica nanoparticles to co-deliver doxorubicin and a P-gp siRNA; this strategy effectively increased the intracellular and intranuclear drug concentration in a resistant cell line. In another example, nanoparticles prepared by self-assembly of polymeric paclitaxel were designed to include (i) P-gp siRNA, (ii) a GSH portion (disulfide bond) for drug release in a reductive environment, and (iii) an aggregation-induced emission fluorogen for diagnostic imaging and polymer stabilization [208]. In vitro, these nanoparticles were capable of inducing cytotoxicity in paclitaxel-resistant ovarian cancer cells. In vivo, pharmacokinetics studies showed that after 8 h, 30% of the nanoparticles were still circulating, thus optimizing the EPR effect. Maximum tumor accumulation was observed at 12 h post injection, which led to delayed tumor growth in three models of paclitaxel-resistant ovarian cancer (subcutaneous xenograft, peritoneal metastasis, and patient-derived xenograft).

Targeting moieties are often included in the formulation. Jia et al. [209] synthesized chitosan oligosaccharide-PEI nanoparticles decorated with folate to target folic acid receptor-overexpressing, multidrug resistant breast cancer cells. A P-gp shRNA was linked via a redox-responsive disulfide bond and paclitaxel was included to pursue a combination strategy. In vitro, this nanoformulation was specifically internalized, it induced silencing of P-gp, and reverted the resistance to paclitaxel. Similarly, Yang et al. produced albumin-based nanoparticles that were targeted to EGFR-overexpressing cancer cells via surface functionalization with cetuximab and embedding doxorubicin and a P-gp siRNA [210]. The nanoparticles had a hydrodynamic diameter of ~175 nm and allowed a sustained release of doxorubicin; in addition, they induced a significant downregulation of P-gp in a breast cancer cell line in vitro. In a mouse model of breast cancer, both doxorubicin and the siRNA were delivered to the tumor, leading to >50% inhibition of cancer growth.

P-gp, or other efflux pumps, may be targeted by miRNAs, as described, for example, in the study by Wang et al. [211]. The authors prepared RNA nanoparticles decorated with three copies of asialoglycoprotein receptor ligands (to target cancer cells), miR-122 (to silence efflux pumps), and 24 molecules of paclitaxel. The efficacy of these nanoparticles in circumventing drug resistance was validated both in vitro and in vivo in models of hepatocellular carcinoma. A similar approach was applied in the design of silica/gold nanoparticles decorated with hyaluronic acid and embedded miR-let-7a and paclitaxel [212]. In a model of drug-resistant ovarian cancer, treatment with these nanoparticles led to the downregulation of P-gp, induction of apoptosis (in vitro), and a marked delay in tumor growth (in vivo).

Yuan et al. aimed to inhibit P-gp indirectly, by silencing its epigenetic regulator, general control non-repressed 5 (GCN5) [213]. The authors prepared mesoporous silica nanoparticles, including a pH/redox responsive element (a disulfide-containing polymer), decorated with hyaluronic acid for tumor targeting, and coloaded with doxorubicin and GCN5 siRNA. These nanoparticles restored drug sensitivity in CD44-overexpressing cancer cells in vitro, and inhibited tumor growth by 77% in vivo while eliminating the systemic toxicity of doxorubicin. With a similar intention, Xiao et al. [214] engineered docetaxel-PLGA nanoparticles to deliver a siRNA against colon cancer-associated transcript 2 (CCAT2), a long non-coding RNA (lncRNA) involved in cancer cell proliferation, invasion, and metastasis [215]. The nanoparticles were functionalized with transferrin peptides for tumor cell targeting, resulting in a final size of ~97 nm. This coformulation proved more effective than nanoparticles loaded with docetaxel only, resulting in good targeting and antitumor activity both in vitro and in vivo, including the downregulation of both P-gp and multidrug-resistance-associated protein 1 (MRP1) in lung cancer cells.

Pharmaceutical inhibitors of P-gp have also been included in therapeutic nanoparticles to overcome multidrug resistance. Wand et al. [216] engineered carbon “nano-onion”/silica nanoparticles of 28 nm, loaded with both a P-gp small-molecule inhibitor (HM30181A) and doxorubicin. The shell was coated with fucoidan to target the surface of tumor endothelial cells. Brief irradiation with low-power NIR light led to a photothermal effect and release of the drug payload, with the consequent inhibition of P-gp and potentiated cytotoxic effect of paclitaxel both in vitro and in vivo in three multidrug-resistant ovarian carcinoma cell lines. Gote et al. [217] prepared micelles made of hyaluronic acid-PLGA (responsive to reductive conditions due to the presence of a disulfide bond) and loaded with paclitaxel and ritonavir, another small-molecule inhibitor of P-gp. The uptake and cytotoxic activity of these nanomicelles were tested in vitro on two breast cancer cell lines, and compared with non-tumor cells, showing selective induction of apoptosis in cancer cells.

### 4.2. Tackling Multidrug Resistance by Nanoparticle-Mediated Codelivery of Different Chemotherapeutic Drugs

Combining different drugs is a widely exploited method that may improve therapeutic efficacy even in multidrug resistant settings.

For example, liposomes coloaded with paclitaxel and doxorubicin (ratio 1:10) were validated for their efficacy and safety in Balb/c mice [218]. Several tests were performed, including electrocardiographic exams showing that, in comparison to the same doses of carrier-free drugs, administration of the liposomal formulation led to (i) a higher LD50 (lethal dose for 50% of animals; 28.9–34.7 mg/kg versus 20.8–23.1 mg/kg), (ii) lower cardiac toxicity, and (iii) a survival rate that was three times higher. This study supported the use of nanoparticle-loaded chemotherapies in high doses and in combination.

Several applications on this topic have been recently published. Logan et al. [219] synthesized nanobubbles based on DSPE-PEG and phospholipid-linked gemcitabine prodrug exposed on the external side and released intracellularly by the action of phospholipase C or D. The resultant very small-sized nanobubbles (~2 nm) were loaded with paclitaxel in the internal phase. In vitro testing on pancreatic cancer cell lines and spheroids showed synergistic cytotoxic effects of the drug combination compared with empty gemcitabine-lipid nanobubbles. Accordingly, the nanobubble formulation was more efficient than free gemcitabine and paclitaxel in delaying tumor growth in vivo in a mouse model of pancreatic cancer.

A codelivery of paclitaxel and gemcitabine to pancreatic cancer cells was also achieved by their encapsulation into liposomes targeted to α_5_β_1_ integrin via the fibronectin mimicking peptide ligand PR_b [220]. The liposomes were further included into a poly(δ-valerolactone-coD,L-lactide)-b-PEG-b-poly(δ-valerolactone-coD,L-lactide) hydrogel for sustained release of the drugs. In vitro, this formulation was validated on both cultures and spheroids as being more efficient than non-targeted liposomes or free drugs [221].

In a work by Zhao et al. [222], paclitaxel and niclosamide were individually included into nanocrystals based on TPGS and Poloxamer 188, respectively. The nanocrystals had sizes of 163.18 ± 3.27 and 146.48 ± 3.22, respectively, and were incorporated in a PLGA-PEG-PLGA hydrogel to obtain an injectable formulation. In vitro, both drugs were released in a slow and sustained mode, as assessed over a period of 14 days. Cell uptake was higher for the hydrogel–nanocrystal formulations than the free drugs, although no improvement in tumor cell killing was observed. Instead, in vivo, a single coadministration of niclosamide and a paclitaxel nanocrystal hydrogel resulted in synergistic effects, with an inhibition of tumor growth by ~68.8%, decreased numbers of proliferating cancer cells, and increased apoptosis.

In another formulation, paclitaxel and salinomycin were included into lipid nanocapsules to achieve a therapeutic effect on breast cancer cells and cancer stem cells, respectively [223]. The derived nanoparticles had a hydrodynamic diameter of 90 ± 5 nm, with a drug loading efficiency of >98%. In vitro experiments on cell lines and 3D tumor mammospheres demonstrated the efficacy of paclitaxel/salinomycin codelivery in terms of cytotoxicity and induction of apoptosis.

Tarannum et al. [224] synthesized mesoporous silica nanoparticles, in which cisplatin and gemcitabin were linked to the core and the surface, respectively, via pH-responsive covalent bonds. The nanoparticles were decorated with an antibody against mucin 1 (TAB004) for tumor targeting. Biodistribution and toxicity studies were performed in healthy mice with encouraging results. Therapeutic studies were performed in a syngeneic mouse model of mucin 1-expressing pancreatic adenocarcinoma, showing tumor-specific accumulation and pronounced inhibition of tumor growth.

### 4.3. Tackling Multidrug Resistance by Nanoparticle-Mediated Delivery of Natural Compounds or Their Derivatives

Several natural compounds and their derivatives are currently explored as anticancer agents, and some of them have been tested in codelivery nanosystems.

Among these, curcumin is now emerging as promising agent for the treatment of multidrug resistant cancers, due to its inhibitory effect on P-gp, as well as for its proapoptotic effects on tumor cells. In a recent work [225], curcumin was coloaded with paclitaxel into the core of PEG-PLGA nanoparticles functionalized with cRGD for tumor targeting, and a NIR cyanine dye (Cy5.5) was incorporated for fluorescent imaging. The efficacy of this formulation was validated both in vitro and in vivo in a mouse model of mammary carcinoma, showing (i) a consistent decrement in the IC50 values of the encapsulated drugs compared to the free drug (0.11 versus 1.53 for paclitaxel; 1.13 versus 45.00 for curcumin); (ii) a slow release within the first 2 h and a quicker release of both drugs over 9 h at an acidic pH (~95% at pH 5.5); (iii) a targeted cellular uptake and synergistic cytotoxic effect in vitro; (iv) accumulation at tumor sites and a synergistic antitumor effect in vivo.

Another natural compound, resveratrol, was coloaded with paclitaxel in polymeric nanoparticles based on Soluplus^®^ (polyvinyl caprolactam-polyvinyl acetate-PEG) and D-α-tocopheryl PEG succinate [226]. The nanoparticles, at sizes of 102.9 ± 0.17 nm, allowed a slow drug release (~25%) in the first 3 h, and a sustained release up to 60–70% thereafter. The cytotoxic efficacy of the combined formulation was 2-fold higher than the free drugs, with synergistic behavior. In vivo, positive pharmacokinetic parameters were associated with 4-fold brain accumulation compared with the free drugs.

Etoposide (a semisynthetic derivative of podophyllotoxin from *Podophyllum peltatum*) and paclitaxel were coloaded into mPEG-PLGA nanoparticles and tested in models of glioblastoma in vitro and in vivo [227]. Although the decreased IC50 of nanoparticle-delivered versus free drugs was not impressive, in vivo, the paclitaxel/etoposide-nanoparticles demonstrated (i) good safety profiles in terms of hemolysis, blood clotting, or platelet aggregation and (ii) potential curative attributes in a group of responder animals, as evaluated by MRIs up to 72 days post tumor implant, at which time no animal was alive in the other treatment arms (namely, vehicle, free paclitaxel, free etoposide, free paclitaxel and free etoposide, paclitaxel-nanoparticle and etoposide-nanoparticle).

Naringenin (a flavanone from grapefruit) was loaded along with paclitaxel in solid lipid nanoparticles and evaluated as potential therapeutic approach for glioblastoma multiforme [228]. The nanoparticles, prepared through a systematic product development strategy to optimize formulations and synthesis protocols, were assembled from Percirol^®^ ATO5 (solid lipid), Dynasan^®^ 114 (surfactant), Lutrol^®^ F188 (stabilizer), and DSPE-mPEG in the presence of both drugs. Surface decoration with cRGD was included for tumor targeting. The derived nanoparticles had a size of 129 nm, drug entrapment efficiency of >80%, and a loading efficiency of >7%. Drug release, cancer cell uptake/cytotoxicity (in vitro), and pharmacokinetics (in vivo) resulted in a better performance compared with the free drugs.

Combretastatin (a natural phenol from *Combretum caffrum* and a vascular disrupting agent), verteporfin (an inhibitor of the Hippo/yes-associated protein, YAP, pathway), and paclitaxel were coloaded into nanoparticles based on lecithin, DSPE-PEG, and PLGA [229]. The authors tested these nanoparticles in vitro, demonstrating an inhibitory effect on the viability and migration of triple-negative breast cancer cells, as well as the reversal of paclitaxel- and combretastatin-mediated implementation of the cancer stem cell compartment. Studies ex vivo (patient-derived organoid cultures) and in vivo (patient-derived xenograft models) confirmed the synergistic efficacy of the triple-drug formulation, with a remarkable delay in tumor growth.

### 4.4. Tackling Multidrug Resistance by Nanoparticle-Mediated Delivery of Small-Molecule Inhibitors or Nucleic Acids

In other applications, small-molecule chemicals that act as inhibitors of tyrosine kinases or other cell transducers have been included in nanoparticle formulations to overcome multidrug resistance.

17-AAG (HSP90 inhibitor) was coloaded with paclitaxel in poly-ε-caprolactone nanoparticles [230] and tested in vitro on breast cancer cell lines, demonstrating a synergistic effect. Chemmalar et al. [231] synthesized calcium carbonate nanoparticles from the ark clam *Anadara granosa*, packed with gefitinib and/or paclitaxel. The deriving mesoporous nanoparticles were spherical, with sizes ranging from 63.9 ± 22.3 to 87.2 ± 26.7 nm and a negative charge. In mild acidic conditions, the carbonate shell increased its solubility, leading to slow (<30% over 100 h) and sustained drug release. In another work [232], carfilzomib (a proteasome inhibitor) was coloaded with paclitaxel (ratio 1:2) in human serum albumin nanocarriers by a self-assembly procedure, giving nanoparticles a size of <150 nm and a negative charge. In a mouse model of pancreatic carcinoma, the dual-loaded nanoparticles exhibited a synergistic effect compared with nanoparticles loaded with each drug, despite pharmacokinetic and biodistribution profiles similar to the controls.

Finally, nucleic acids have also been incorporated into nanoparticles for therapeutic applications that overcome multidrug resistance. Multifunctional micelles based on PEI-PLA-lipoic acid copolymers were loaded with paclitaxel (via hydrophobic interaction) and a siRNA against STAT3 (via electrostatic interaction) [233]. The final nanoparticle was coated with hyaluronic acid to confer tumor-targeting properties. Upon selective binding to CD44-expressing cancer cells, nanoparticles underwent endocytosis, and the hyaluronic acid coating was degraded in the lysosomal compartment. The effect of STAT3 siRNA and paclitaxel was confirmed in vitro and in a model of metastatic mammary carcinoma. PEI-PLGA nanoparticles loaded with paclitaxel and a siRNA against VEGF were tested in the same model with promising results, demonstrating the efficacy of concomitant anti-tumor and anti-angiogenic therapy [234]. Similarly, Du et al. developed a liposome-based system including paclitaxel and a siRNA against twinfilin 1, a protein involved in epithelial-to-mesenchymal transition in cancer progression [235]. This liposome was specifically designed to cross the blood–brain barrier and target brain metastases via a surface functionalization with a BRBP1 peptide [236]. The authors confirmed that the coformulation of paclitaxel and twinfilin 1 siRNA potentiated the antitumor effect both in vitro and in a model of drug-resistant metastatic breast cancer.

Chen et al. [237] synthesized nanoparticles for the codelivery of paclitaxel and a miRNA with anti-oncogenic effects (miR-124). The nanoparticles were based on mPEG-hyaluronic acid-phosphocholine polymers containing a disulfide bond (a GSH-responsive element). The intracellular detachment of hyaluronic acid was followed by the release of both paclitaxel and miR-124, with consequent antitumor activity in vitro and in vivo. As a final example, Majumder et al. [238] prepared lipid nanocarriers based on trilaurin, α-tocopherol, DSPC, and DSPE-PEG, with the latter covalently linked to LHRH as a tumor-targeting moiety. The nanocarrier included paclitaxel, gefitinib, an EGFR siRNA, and/or rhodamine as an imaging agent. In vitro, this formulation was efficiently taken up by lung cancer cells, with a consequent reduction in EGFR activation, and 5–10 times higher cancer cell cytotoxicity compared with the free drugs.

**Figure 3:**
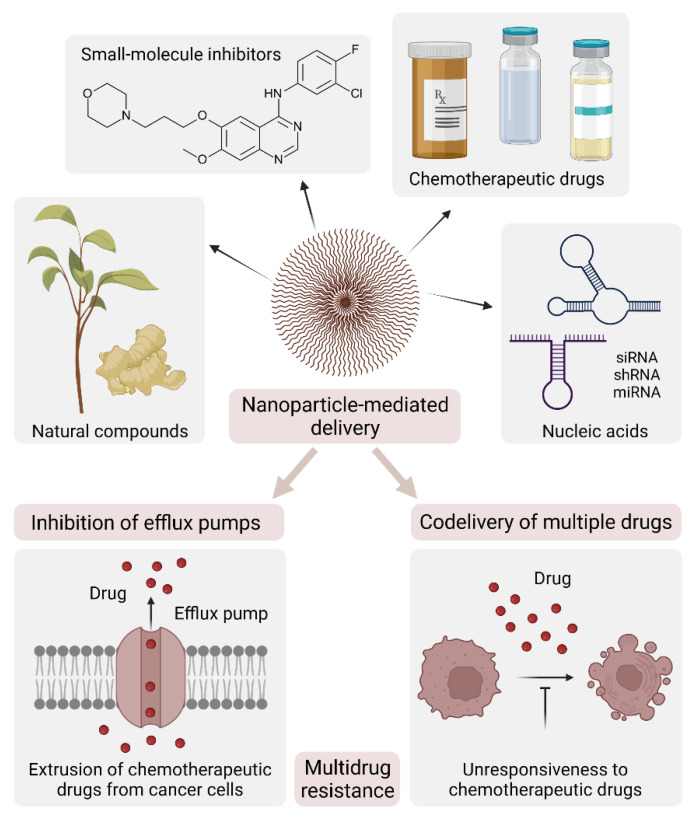
Strategies to overcome multidrug resistance by the use of nanoparticles. Common mechanisms of resistance include (i) an increased exit of the drug via specific pumps and (ii) primary or acquired inability of the drug to induce cytotoxicity due to altered signaling pathways. The efficacy of chemotherapeutic drugs can be restored by delivering different classes of active compounds in order to (i) inhibit the synthesis and/or the activity of an efflux pump and (ii) simultaneously block different intracell pathways, respectively. Created with BioRender.com.

The strategies to overcome multidrug resistance with the use of nanoparticles are illustrated in Figure 3, and the described nanoparticles are summarized in Table 10.

## 5. Nanoparticle-Based Cancer Vaccines

Cancer cells express peculiar antigens that are recognized by circulating clones of B cells, leading to the production of specific antibodies (innate immunity, humoral). Cancer antigens are also processed by antigen-presenting cells (e.g., dendritic cells), and exposed to the major histocompatibility complex (MHC). When the antigen presents on MHC-I, it recruits CD8+ T cells and primes them to differentiate into cytotoxic T lymphocytes (CTLs) that kill cancer cells (adaptive immunity, cell-mediated). When it presents on MHC-II, it recruits CD4+ T cells and induces their differentiation in T helper lymphocytes that potentiate B-cell response. The activation of T lymphocytes is regulated by coreceptors with a stimulatory or inhibitory effect. Among these, CTL-associated protein 4 (CTLA-4) and programmed cell death 1 (PD-1), or its ligand (PD-L1), hinder T cell activation, and are therefore defined checkpoint inhibitors. Upon cancer progression, different immune escape mechanisms take place, including the onset of tolerance to cancer-associated antigens (especially when similar to natural antigens) and overexpression of CTLA-4, PD-1, or PD-L1, to inhibit the cytotoxic effect of CTLs on tumor cells.

Current approaches aim to design vaccines that exploit cancer antigens to develop effective targeted immunotherapies. Nanoparticles are appropriate for this purpose, since they can act as both carriers—to deliver high payloads of cancer antigens and/or immune checkpoint inhibitors, simultaneously preventing their degradation and ensuring a controlled release—and adjuvants—to promote/amplify the interaction with antigen presenting cells. The concept of applying nanotechnology to vaccine development is not new. In 1998, Gu et al. designed nanoparticles based on mannan or pullulan, modified with cholesteryl groups, and including an antigenic portion of HER2 [239]. These nanoparticles triggered activation of CD8+ T cells into CTLs with activity against HER2-expressing cells. In vivo, vaccination using the HER2-carrying nanoparticles induced the production of specific antibodies and tumor rejection in mice xenografted with HER2-expressing cancer cells. Here, we examine approaches that tackle different issues in the optimization of nanovaccines and provide elegant solutions.

### 5.1. Strategies to Optimize Nanovaccine Formulations

#### 5.1.1. Coencapsulation of Adjuvants and Antigens

CpG oligonucleotides are broadly utilized adjuvants that stimulate the activity of antigen-presenting cells via activation of toll-like receptors (TLR). Nanoparticles based on PEI-CpG complexes have been validated as efficient carriers, with good internalization and immune-stimulation properties. In an immunocompetent murine model of melanoma, these nanoparticles triggered both humoral and cellular responses, and inhibited tumor growth [240]. An interesting application of this concept is described by Nam et al. [241]. In this case, a PEI-PEG polymer was covalently conjugated with a selection of cancer neoantigens—Adpgk peptide (identified in the MC38 mouse model of colon carcinoma), MHC I-restricted M27 peptide, and/or MHC II-restricted M30 peptide (both identified in the B16F10 mouse model of melanoma)—and successively self-assembled with the CpG adjuvant. The derived nanoparticles triggered a stimulation of antigen-presenting cells and activation of CD8+ T cells. When administered intratumorally, they induced a sustained (over 3 weeks) CD8+ T cell response, eradicated tumors in 60% of mice bearing MC38 xenografts (nanovaccines with the Adpgk antigen), and inhibited lung metastases in mice bearing B16F10 xenografts (nanovaccines with M27/M30 antigens).

Nanoparticles carrying model antigens (like ovalbumin) have been tested to implement the coencapsulation of adjuvants and peptide antigens. This is a difficult task due to the diverse chemical properties of these two classes of compounds. Van Lysebetten et al. [242] linked an ovalbumin antigen (CSSSIINFEKL peptide), or the adjuvant imidazoquinoline (an agonist of TLR7/8), to poly(l-glutamic acid). The deriving conjugates were condensed with an ionizable (cationic) lipid and structural lipids, and the final nanoparticles were obtained by adding a PEG coating. A T-cell response was achieved both in vitro and in vivo after subcutaneous or intravenous vaccination. In another work, PLGA-based nanoparticles were prepared with the inclusion of the double-stranded RNA adjuvant Riboxxim (an agonist of TLR3) and ovalbumin [243]. In vitro, this formulation triggered the activation of human and murine antigen-presenting cells. In vivo, treatment with PLGA-Riboxxim nanoparticles carrying the ovalbumin antigen prevented tumor grafting (prophylactic approach), led to the regression of established tumors, increased animal survival, and reduced the number of lung metastases in mouse models of thymoma and metastatic melanoma (therapeutic approach). The effect was potentiated by inclusion in the treatment regimen of an anti-CTLA-4 antibody for concomitant immunotherapy. This system appears promising in terms of future clinical translation.

#### 5.1.2. Inclusion of Multiple Antigen Peptides with Different Chemical Properties

A smart way to overcome the issue of including peptide antigens with different polarity has been reported by Shi et al. [244]. They fused two well-characterized cancer antigens, namely, the melanoma-specific MAGE-A1 (hydrophilic) and the esophageal squamous cell carcinoma-specific NY-ESO-1 (hydrophobic), with a dipeptide linker substrate of cathepsin B. These conjugates self-assembled and were incorporated into a lipid bilayer derived from erythrocyte membranes with the addition of mannose residues. The derived nanoparticles, at a size of ~155 nm, were specifically recognized by dendritic cells through mannose receptors, and the antigenic peptides were separated in the cytoplasm by the action of cathepsin B. As a consequence, cellular immunity was triggered in vitro. When tested in two xenograft models of breast carcinoma, a substantial growth inhibition (up to 90%) of established tumors was achieved.

Another crucial step is the inclusion of multiple antigens, which is even better with patient specific cancer neoantigens. Although this feature has the potential to render a vaccine curative, it is also problematic from both a chemical and a genetic point of view. A couple of brilliant approaches have recently been reported. Hu et al. [245] designed a flash nano complexation-based procedure for coating different nanocarriers (PLGA, PEI-DNA, silica nanoparticles with or without pores of different sizes) with cancer cell-derived membranes (exposing cancer-specific antigens). Among the several formulations that have been prepared and tested, the authors selected big-pore mesoporous silica nanoparticles coated with membranes from B16F10 melanoma cells for further investigation. As an adjuvant, they used CpG oligonucleotides. When injected in mice, this nanovaccine formulation reached the lymph node compartment after 1 h, with a peak at 12 h, and a decrease after 24 h. In experimental tumor models, the nanovaccine was efficient in both prophylactic (1 dose/week starting 3 weeks before tumor grafting) and therapeutic settings (doses at day 2, 4, and 7 post tumor grafting, in combination with an anti-CTLA-4 antibody), where ~60% and ~80% of the animals survived (up to 150 days, likely cured), respectively.

Similarly, Xiong et al. [246] synthesized PLGA-based nanoparticles coated with membranes from doxorubicin-treated breast cancer cells, a situation in which cells expose neoantigens on their surface while undergoing the so-called immunogenic death. Calcinetin, a protein that binds dendritic cells and induces receptor-mediated uptake, was represented at high levels in the membrane coating. As an adjuvant, the authors included imiquimod in the nanoparticle core, and its release was characterized as improved at pH 6.8 (reproducing the tumor microenvironment) and maximized at pH 5.0 (reproducing lysosomal conditions). In vivo, this formulation efficiently reduced the growth of established tumors (67.8% inhibition). In prophylactic experiments, tumor inhibition was up to 70.80% and was accompanied by stimulation of specific T cells.

The strategies discussed above are summarized in Table 11.

### 5.2. Examples of Antigens in Successful Preclinical Nanovaccines

#### 5.2.1. Melanoma Antigens

Melanoma-derived peptides are amongst the most exploited as cancer-specific antigens in nanovaccines. A recent study explores six alternative protocols to include antigenic melanoma peptides into nanomicelles based on PEG-b-poly(2-hexamethyleneimino ethyl methacrylate) (PC7A nanoparticles) [247]. The authors tested a panel of peptides, including B16F10-derived antigens (Gp100_21–41_, Trp1_214–237_), and neoantigens (Obsl1_T1764M_, Pbk_V145D_, Tnpo3_G504A_). Trp1 and Tnpo3 were efficiently encapsulated with most protocols and released in mild acidic conditions (pH 6.4). A nanovaccine including both peptides had significant antitumor effects in vivo. Tsai et al. [248] investigated the use of polyplexes obtained with the electrostatic interaction of Trp1 melanoma antigen fused to an arginine stretch (cationic portion) and CpG (anionic portion). The authors evaluated different ratios of the two components (Trp2:CpG from 1:5 to 10:1), obtaining nanoparticles with different sizes and tunable surface charges. In the polyplexes, CpG oligonucleotide was protected from degradation and it potentiated the action of dendritic cells with a maximum at the 5:1 ratio. CD8+ T cells were also differentially activated depending on the component ratios. In addition, the authors show that the efficiency of antigen uptake by dendritic cells depends on the number of arginine residues in the cationic portion. Although the reported results of in vivo therapeutic vaccinations are modest, this work nevertheless gives important clues on how to implement specific immune responses.

In an outstanding study, Baharom et al. explore different administration routes and doses of nanomicelles including both Reps1 (neoantigen from the MC38 melanoma model) and an imidazoquinoline-based TLR7/8a agonist (adjuvant) [249]. They observed that subcutaneous vaccination was more efficient than intravenous vaccination in priming CD8+ T cells (20-fold higher), and in inducing the expression of effector versus stem-like genes. In prophylactic experiments, subcutaneous administration of the nanovaccine resulted in controlled tumor growth and extended survival, either alone or in combination with an anti-PD-L1 antibody. Instead, in therapeutic experiments, the intravenous route of administration was successful in reducing tumor growth and was curative for 100% of the mice, whereas the subcutaneous route was not effective. These results appear crucial for the rational design of cancer vaccines with different purposes (preventive versus curative).

#### 5.2.2. Mucin Antigens

Other tumor-specific antigens for vaccine development include mucins, membrane glycoproteins that are often overexpressed, and present different patterns of glycosylation in cancer cells, as previously discussed for mucin 1. Recent papers report (i) gold nanoparticles linked with mucin 1 glycopeptide as an antigen and the T cell activator α-galactosyl ceramide as an adjuvant [250]; (ii) gold nanoparticles associated with a polysaccharide as targeting moiety to dectin-1 on dendritic cells, ovalbumin and a specific glycosylated form of mucin 4 including the Thomsen–Friedenreich disaccharide as antigens, and Sigma Adjuvant System or TiterMax Gold as adjuvants [251]; (iii) amphiphilic polyanhydride copolymer nanoparticles, as both a carrier and an adjuvant, loaded with mucin 4β as an antigen [252].

#### 5.2.3. Miscellaneous Antigens

Cancer nanovaccines exploiting additional antigens are also in the phase of preclinical study. Here are some very recent examples: (i) PLGA nanoparticles including NY-ESO-1-derived peptides (amino acids 85–111, 117–143, and 157–165) as antigens and the α-galactosyl ceramide analog IMM60 as an adjuvant [253]; (ii) Vx3-alumina nanoparticles (where Vx3 is an ubiquitin-binding protein) carrying ubiquitinated proteins from epirubicin-treated multi-drug-resistant stem-like breast cancer cells as antigens and a stimulator of interferon genes (STING) agonist as an adjuvant [254]; (iii) manganese-doped silica nanoparticles as both a carrier and an adjuvant and GF001 peptide from human papilloma virus 16 (HPV16) protein E7 as an antigen [255]; and (iv) ultra-small polymeric nanoparticles conjugated to an HPV16 E7 long-peptide as an antigen and CpG as an adjuvant [256]. In all these cases, in vitro and in vivo experiments confirmed the activation of immune responses in therapeutic and/or prophylactic settings, in some cases with a curative outcome in mouse models.

The nanovaccines described in this section are recapitulated in Table 12.

### 5.3. Multimodal Nanovaccines

#### 5.3.1. On-Demand Activation of the Nanovaccine at the Tumor Site

Advanced nanovaccines provide further functions, including the possibility to activate the adjuvant and/or the antigen exactly at the tumor site and with precise timing. One such formulation is based on an amphiphilic dendrimer of a 2-nitroimidazole derivative (hydrophobic portion) and a poly-lysine dendron (hydrophilic portion), self-assembled to form nanomicelles of 28.6 ± 2.26 nm. The nanomicelles were loaded with a photosensitizer, chlorin e6, to obtain nanoparticles of 43.22 ± 3.40 nm, which accumulated at the tumor site, allowing a precise NIR laser irradiation with a consequent release of chlorin e6 and generation of ROS. The strategy implies that cancer cells, upon hypoxia and ROS-induced damage, release specific antigens to induce immune responses. At the same time, the change in redox conditions converts 2-nitroimidazole to 2-aminoimidazole, which is a potent adjuvant. This approach proved effective in vivo, in terms of both lymphocyte stimulation and delayed tumor growth in models of breast and colon carcinoma. Interestingly, this light-inducible nanovaccine was active not only on the irradiated tumor mass, but also on additional tumors (abscopal effect), as observed in a model of bilateral xenografts, demonstrating a successful triggering of systemic immunity [257].

Another smart application utilizes a nanovaccine incorporated into a hydrogel [258]. Upon treatment with ultrasounds, the hydrogel liquifies and the nanovaccine is released. When treatment is suspended, the gel returns to its original consistency and the cycle can be repeated several times, thus allowing iterated vaccine boosts with a single subcutaneous injection. With this approach, the authors report administration of PLGA-based nanoparticles coloaded with an ovalbumin peptide as an antigen and imiquimod as an adjuvant, which were included in an oligo(ethylene glycol) methacrylate hydrogel [259]. Mice were administered the gel-nanovaccine formulation and received ultrasound treatments every other day for 14 days; at day 21, they were implanted orthotopically in metastatic breast cancer cells. Promisingly, a substantial fraction (42.8%) of mice in this treatment group rejected the tumor and were still alive after 240 days.

#### 5.3.2. Nanovaccines That Combine Multiple Steps and/or Functions

Zhang et al. designed a nanovaccine that works in successive steps [260]. They assembled modular nanoparticles of 220.5 nm, based on poly-[(N-2-hydroxyethyl)-aspartamide]-Pt(IV)/β-cyclodextrin (cisplatin prodrug), CpG/PAMAM-thioketal-adamantane (adjuvant/carrier), and mPEG-thioketal-adamantane (further carrier component), which accumulated at the tumor site when injected in animal models. As a first step, the thioketal links are cleaved in the tumor microenvironment in response to high levels of ROS. This leads to the second step, wherein the released cisplatin kills cancer cells and CpG/PAMAM activates lymph node-resident dendritic cells, thus potentiating the immune response to cancer antigens. These steps were validated by in vitro assays and confirmed by the outcome of in vivo therapeutic experiments, where the multistep nanovaccine led to 73% inhibition of tumor growth and activation of dendritic cells, CD4+ and CD8+ T cells. When combined with an anti PD-L1 antibody, the vaccine cured 40% of the animals.

Modern nanovaccines may also combine other anticancer treatments. For example, Gao et al. [261] describe dextran-based nanoparticles including indocyanine green (agent for photothermal therapy) in addition to an albumin-derived peptide (antigen) and imiquimod (adjuvant). Upon NIR light irradiation, indocyanine green causes hyperthermia to kill tumor cells. Mice treated with this formulation were completely protected from grafting with mouse thymoma cells, which could be the premise of future clinical applications.

Zhang et al. [262] prepared polydopamine–hyaluronic acid nanoparticles coloaded with imiquimod and doxorubicin and characterized by high photothermal conversion efficiency. For in vivo administration, the nanoparticles were included into a thermosensitive hydrogel to allow repeated photothermal ablation by irradiation of the tumor mass with NIR light. The combined effect of doxorubicin and hyperthermia resulted in cancer cell death and release of specific neoantigens. The immune response was potentiated by the presence of the adjuvant, with consequent maturation of dendritic cells and accumulation of T cell populations both in the lymph nodes (memory lymphocytes) and in the spleen (CTLs). This treatment was curative at 20 days after orthotopic implant of breast cancer cells in immunocompetent mice.

In another work, radiotherapy and immunotherapy were combined, exploiting mesoporous silica nanoparticles of 104.22 nm carrying ovalbumin as a model antigen [263]. The authors set up bilateral tumors by grafting a syngeneic hepatocellular carcinoma model into each leg of the mice. Radiotherapy directed to one of the tumors had no effect on the other. Instead, administration of the nanovaccine into the irradiated tumor, inhibited the growth of both tumors, thus inducing an abscopal effect. The nanovaccine enhanced CTL recruitment and inhibited regulatory T cell infiltration in both sites, thus potentiating the antitumor immune response. When incubated with supernatants of irradiated hepatocellular carcinoma cells (undergoing immunogenic death) prior to intratumor injection, the nanovaccine was more effective in synergizing with anti-PD1 immunotherapy compared with the synthesized nanovaccine. The authors explain this result by noting the capacity of mesoporous silica to absorb cancer antigens and trigger the maturation of dendritic cells in tumor-draining lymph nodes.

The peculiarities of these multimodal nanovaccines are summarized in Table 13.

### 5.4. Nanovaccines including Antigen-Coding Nucleic Acids

#### 5.4.1. RNA-Based Nanovaccines

A number of nanovaccines have been designed to deliver nucleic acids and transduce host cells to produce high titers of the antigen(s). In order to optimize RNA encapsulation, Zhang et al. tested fifteen variants of cationic materials derived from PAMAM dendrimers with carbon tails of different lengths (namely, A1-A3, B1-B3, C1-C3, D1-D3, and E1-E3) [264]. These lipid-like materials were coformulated with DSPE-PEG and mRNA coding for an ovalbumin epitope (CD8, OVA_257–264_). Formulation C1 conferred self-adjuvant properties and was the most efficient in stimulating an immune response via activation of TLR4 in vitro. In vivo, the C1 nanovaccine was efficient in both prophylactic and therapeutic trials on syngeneic mouse models of colon carcinoma and melanoma.

In another work, a nanovaccine was prepared, which included an ovalbumin mRNA complexed with a cationic moiety in a palmitic acid-R848 (an agonist of TLR7/8)/PEG nanoparticle [265]. Inclusion of an adjuvant in the formulation resulted in an enhanced vaccine potency, leading to promising results in syngeneic allograft tumor models of lymphoma and prostate cancer, compared with adjuvant-lacking formulations. Both preventive (84% reduction of tumor volume compared to the control) and therapeutic (60% reduction) effects were observed. Nanovaccines have also been prepared with mRNAs from cancer cells. In a very recent report [266], the authors describe PEG-liposomes complexed with polycationic DNA and loaded with total RNA from a murine cell line of colon carcinoma. The resultant nanovaccine, either alone or in combination with oxaliplatin treatment, was administered to mice carrying colon carcinomas from the same cell line and improved the efficacy of chemotherapy.

#### 5.4.2. DNA-Based Nanovaccines

In some cases, the vaccine is based on DNA. For example, a plasmid carrying the ovalbumin-coding DNA was included in calcium phosphate nanoparticles modified with mannose (as a targeting moiety to C-type lectin receptors on the surface of dendritic cells) and bisphosphonate (as a stabilizer) [267]. This nanovaccine was efficient in inducing T-cell responses and in preventing tumor grafting in a syngeneic mouse model of thymoma, leading to a complete rejection in 4 out of 5 mice. In another work of the same group [268], calcium phosphate nanoparticles were synthesized, including an ovalbumin DNA vaccine and ATP as both a stabilizer and an adjuvant. This formulation provided improved stability and dendritic cell activation.

Zhang et al. [269] prepared nanoparticles by self-assembly of a tumor-targeting peptide (RGD-GGG-K18) (cationic) and a DNA plasmid (anionic) encoding an HPV16 E7 epitope fused to the HSP110 gene. Immune cell activation and prophylactic/therapeutic efficacy were reported. In another multifunctional vaccine, chitosan nanoparticles carried a plasmid for the expression of either an activated factor (L-Myc) or an antigen (carbonic anhydrase) [270]. Intramuscular coadministration of the two formulations resulted in suppression of both primary tumors and lung metastases in a model of renal carcinoma, with an induction of mature dendritic cells in the spleen.

Model cancer nanovaccines based on nucleic acids are recapitulated in Table 14.

### 5.5. Outline of Clinical Trials Involving Nanovaccines

Translation of nanovaccines to clinical studies is not a recent step in cancer immunotherapy; so far, however, the results have been scarce. A model example is the development of anti-mucin vaccines. Based on promising results showing that immunization with mucin peptides generates anti-mucin antibodies and CTL responses in patients, different formulations have been designed and tested in clinical trials, including tecemotide (liposomal nanovaccine), TG4010 (virus-based vaccine), and PANVAC™ (virus-based vaccine). Despite its impressive performance in preclinical settings, tecemotide failed to meet the expected curative potential. An exploratory study on chemotherapy-naïve multiple myeloma patients confirmed a humoral response in 47% of them; however, the biochemical changes observed did not reach the criteria of clinical response [271]. In a randomized double-blind phase III trial on patients with non-small cell lung cancer after chemo- and radiotherapy (the START study), no significant difference in overall survival was observed in the general patient population, although improved survival was present in patients previously treated with concurrent chemoradiotherapy [272]. Further investigation suggested that tecemotide might be beneficial for a subgroup of patients with elevated levels of mucin 1 [273]. Another trial on maintenance tecemotide in patients with non-small cell lung cancer, however, did not confirm the partial efficacy reported in the START study [274]. The introduction of bevacizumab in combination with tecemotide met the endpoint of median progression-free survival and overall survival in a restricted group of patients with partial response/stable disease after chemoradiotherapy and consolidation therapy [275]; however, phase II trials on patients with metastatic colorectal cancer [276] or early breast cancer [277] showed no improvements in progression-free/overall survival.

These results indicate that a great space for improvement remains to be explored. Despite the poor efficacy observed up to now, the preclinical success of nanovaccines supports further efforts to perfectionate formulations, doses, and administration schedules, as well as combinations with other antitumor treatments. In line with this observation, tecemotide was discontinued for the START study applications, but is still available for other clinical trials with different designs.

In order to optimize nanovaccine formulations, a recent study investigated the efficacy of synthetic high-density protein nanodiscs in rodents and non-human primates [278]. The authors compare nanodiscs carrying Adpgk antigens and including different adjuvants, namely, CpG class B, CpG class C (TLR9 agonists), and polyinosinic-polycytidylic acid/carboxymethylcellulose/polylysine (polyICLC, an agonist of TLR3). Among these, polyICLC provided the most efficient activation of CD8+ T cells. This formulation, combined with an anti-PD-1 antibody, was capable of eradicating established tumors of large sizes in a model of colon carcinoma. In *Macaca mulatta*, a similar formulation carrying another antigen (Gag) induced CD8+ T cells and amplified the effect of an adenoviral vaccine. The formulations including the CpG class B and CpG class C adjuvant gave contrasting results in the two species, highlighting the need for a careful choice of each nanovaccine component from the perspective of a clinical translation.

In accordance with these findings, a phase I trial of cholesteryl pullulan nanoparticles carrying the CHP-NY-ESO-1 antigen and including polyICLC as an adjuvant has been just published [279]. In a small cohort of patients with advanced/recurrent esophageal cancer, the vaccine was well-tolerated and an immune response was observed, although there was no tumor response. The authors also performed a preclinical trial in mice challenged with NY-ESO-1-expressing cancer cells, observing that, although single-agent nanovaccines or anti-PD-1 antibodies were poor or not effective, tumor growth was inhibited in animals treated with a combination of the two. This finding, combined with the previous report by Najafabadi et al. suggests a worthy modification which should be included in future clinical trials.

Finally, a phase I clinical study is now recruiting patients to find the optimal dose of PRECIOUS-01, a nanovaccine composed of the NKT activator threitolceramide-6, and the NY-ESO-1 antigen included in PLGA nanoparticle.

## 6. Discussion

A plethora of nanoparticles for drug delivery in oncology are being designed by combining different carrier materials and active agents. Many prototypes are built on FDA-approved components to facilitate future clinical development. Excellent studies provide preclinical proof of superiority versus current chemotherapies in terms of improved antitumor efficacy (even in multidrug resistant tumor settings) and reduced systemic toxicity. Of particular importance is the entrapment of poorly soluble and scarcely bioavailable drugs, which can be concentrated into the nanoparticle, so that the therapeutic payload is both protected from degradation and prevented from acting off-site.

Despite great excitement for the positive preclinical achievements, several drawbacks remain when attempting to translate these nanoplatforms to clinical practice, as we learned from the ThermoDox^®^ and tecemotide cases which were previously examined [23]. Accordingly, ClinicalTrials.gov (accessed on 18 March 2022) reports <50 recruiting/complete interventional trials that investigate new nanoformulations, including NanoPac^®^ (submicron particle paclitaxel), CriPec^®^ (polymeric nanoparticle co-assembled with docetaxel prodrug), EP0057 (polymeric nanoparticle co-assembled with camptothecin prodrug), ABI-009 (albumin-rapamycin), NC-6004 (cisplatin-loaded polymeric nanomicelle), SNB-101 (double core-shell nanomicelle encapsulating SN38), BIND-014 (docetaxel-loaded, PSMA-targeted nanoparticle), C225-ILS-DOX (doxorubicin-loaded, EGFR-targeted liposome), EGEN-001 (biodegradable polymer carrying a plasmid for the expression of human interleukin 12), TKM-080301 (lipid nanoparticle carrying a siRNA against Polo-like kinase 1), MesomiR1 (EGFR-targeted bacterial micelles including miR-16), and INT-1B3 (lipid nanoparticle carrying a modification of miR-193a-3p). Although most of these clinical trials are still open, emerging preliminary results are not impressive.

A main limitation of nanoparticles as drug/vaccine nanocarriers is their retention by healthy organs, particularly the spleen and liver, a feature observed in most studies discussed in the present review. This limitation can be contained by refining the physicochemical features of the nanoparticles; for example, by exposing PEG on their surface to substantially reduce opsonization by the reticuloendothelial system. Another non-secondary limitation relies on the composition and features of the tumor microenvironment, which may influence drug delivery to malignant cells. To overcome this limitation, modern prototypes also include microenvironment-specific features (for example, pH-, redox-, and ROS-responsive drug release), antiangiogenic compounds, and/or immunotherapeutic functions.

Starting >35 years ago, great promise for tumor-directed nanomedicine came from the introduction of “magic bullet”-driven nanocarriers, which are now routinely implemented as increasing numbers of specific peptides, antibodies, aptamers, sugars, and so on, are characterized as targeting moieties. This approach, however, has not been as successful as expected in overpowering the EPR-dictated accumulation of therapeutic nanoparticles at tumor sites. A multivariate analysis on 232 datasets from 2005 to 2015 [280] is enlightening in this regard. This analysis shows that active targeting is indeed more efficient than passive targeting, but the increase is slight (0.9% of the injected dose versus 0.6%), and overall, the numbers are staggeringly low: less than one nanoparticle in a hundred reaches its target tissue. This implies that high amounts of nanoparticles need to be injected to achieve therapeutic doses at the tumor site, which is a fundamental restraint to take into account when thinking of clinical translations—not forgetting the prohibitive production costs of multi-component nanosystems. Targeting tumor blood vessels instead of (or in addition to) cancer cells may provide a better performance, as pioneered by many works from both Ruoslahti’s and Pasqualini/Arap’s groups since the late 1990s [281]. One reason for this lies in the peculiar surface proteome of angiogenic vessels, the so-called vascular zip code, which allows specific binding of ligand-directed circulating molecules [282,283]. A second reason comes from the recent finding that as much as 97% of the nanoparticles enter the tumor tissue via transendothelial migration [31], which would be favored by specific binding and internalization.

## 7. Conclusions

The most recent trend is to produce nanomedicines that are both nature-friendly (with organic and/or biodegradable components) and biocompatible, to minimize the impact on the environment and to advance safe applications for human health. Several nanoparticles include compounds traditionally employed in natural medicine, such as phytochemicals and their derivatives, and/or repurposed drugs. In addition, multiple functions are often incorporated, including additional therapeutic systems (for example, radiotherapy, hyperthermia, ultrasound- or laser-induced drug release) or diagnostic agents. Of particular note is the association between chemotherapy and immunotherapy, a regimen that has led to complete tumor regression in several preclinical models. Together, nanotechnology holds huge promises for improving the way we fight cancer.

## Figures and Tables

**Table 1:** Examples of strategies adopted to optimize drug loading, release, uptake, and delivery.

Carrier	Drug	Strategy	Achievement	Optimized Step	Ref.
Liposome	Doxorubicin + simvastatin	Hydrophilic and hydrophobic portions	Coencapsulation of drugs with opposite solubility	Loading	[11]
Double-emulsion nanoparticle	Doxorubicin + erlotinib	Hydrophilic and Hydrophobic portions	Coencapsulation of drugs with opposite solubility	Loading	[12]
Liposome	Doxorubicin	Different lipid composition and internal pH	Slow, medium, or fast drug release	Release	[13]
Lipid nanoparticle	Doxorubicin	Shell with different thickness	Slow, tunable (thickness) release	Release	[14]
Polymeric nanomicelle	Doxorubicin	Hydrophobic and cationic portions	Slow, sustained release	Release	[15]
Calcium carbonate microcapsule	Doxorubicin	Alternate layers of polyanions and polycations	Slow, tunable release	Release	[16]
Polymeric nanomicelle	Camptothecin	Different backbone length	Fast/high or slow/low uptake	Uptake	[17]
Amphiphilic nanoparticle	Doxorubicin	Redox/pH-responsive	Activable intracellular unloading	Delivery	[18]
Vitamicelle	Doxorubicin	Redox/pH-responsive	Activable intracellular unloading	Delivery	[19]
Lipid nanomicelle	Doxorubicin	Artificial cytosol	Favored intracellular unloading	Delivery	[20]

**Table 2:** Examples of nanoparticles responsive to biochemical features of the tumor microenvironment.

Carrier	Drug	Modality	Strategy	Responsive to	Ref.
Starch	Doxorubicin	Unbound drug	Ionizable portion	Low pH	[33]
Albumin	Doxorubicin	Unbound drug	Ionizable portion	Low pH	[34]
PEG-CaCO_3_ nanoparticle	Doxorubicin	Unbound drug	Degradation	Low pH	[35]
Nanocapsule with ferrocene	Paclitaxel	Unbound drug	Ferrocene oxidation, surface charges	Presence of ROS	[36]
Carbon nanotube	Doxorubicin + paclitaxel	Unbound drug	Ionizable portion, degradation	Low pH	[37]
PEG-drug	Doxorubicin	Prodrug	Ionizable portion	Low pH	[38]
PEG-drug, lecithin	Cisplatin + paclitaxel	Prodrug	Ionizable portion, disulfide bonds	Low pH, reductive conditions	[39]
Drug dimer-F127, DSPE-PEG, albumin, or Fe-tannic acid	Paclitaxel	Prodrug	Diselenide bond	Reductive conditions	[40]
Drug-dextran	Paclitaxel	Prodrug	Disulfide bond	Reductive conditions	[41]
Drug-tetramethyl pyrazine	Paclitaxel	Prodrug	Disulfide bond	Reductive conditions	[42]

**Table 3:** Examples of nanoparticles responsive to biochemical features of the tumor microenvironment and including additional functions.

Carrier	Drug	Additional Functions	Mechanism	Responsive to	Ref.
Upconversion nanoparticle-albumin	Doxorubicin Chlorin 6, Fe^2+^	Photodynamic Chemodynamic	Production of ROS by NIR and H_2_O_2_	NIR irradiation Low pH	[43]
Copolymer nanoparticle	Doxorubicin prodrug Chlorin 6	Photodynamic Chemodynamic	Production of ROS by NIR	NIR irradiation Reductive conditions	[44]
Nano-donuts	Doxorubicin Prussian Blue Fe^3+^ ion	Chemodynamic Photothermal MRI	Production of ROS by H_2_O_2_ Induction of hyperthermia	NIR irradiation, Presence of ROS	[45]
PEG-PEI-based nanoparticle	Docetaxel IR825	Photothermal	Induction of hyperthermia	NIR irradiation, Low pH	[46]
Semiconducting polymer	Doxorubicin prodrug	Photothermal	Induction of hyperthermia	NIR irradiation, Low pH	[47]
Dendrimer/ Lentinan nanoparticle	Paclitaxel	Photodynamic	Production of ROS by NIR	NIR irradiation, Low pH, Reductive conditions, Presence of ROS	[48]

**Table 4:** Examples of mAb-functionalized nanoparticles for targeted drug delivery.

Carrier	Drug	Antibody	Target	Tumor Model	Ref.
PEG-nanomicelle	Taxol	α-nucleosome	Nucleosome	Lewis lung carcinoma (in vitro and in vivo)	[51]
Liposome	Paclitaxel + salinomycin	Clone 2C5	Nucleolin	Breast cancer (in vitro)	[52]
Nanorod	Paclitaxel	Trastuzumab	HER2	Breast cancer (in vitro)	[55]
Gold nanorod/ PLGA-PEG nanoparticle	Paclitaxel	Trastuzumab	HER2	Breast cancer (in vitro)	[56]
Chitosan/PLGA nanoparticle	Doxorubicin	Trastuzumab	HER2	Breast cancer (in vitro)	[57]
Fucoidan/chitosan nanoparticle	Gemcitabine	Clone 7H3L20	HER2	Breast cancer (in vitro and in vivo)	[58]
Gold nanoparticle		Cetuximab	EGFR	Colorectal cancer (in vitro)	[59]
PLGA-PEG nanoparticle	5-FU	α- EGFR	EGFR	Colorectal cancer (in vitro)	[60]
Chitosan/PEG nanocapsule	Docetaxel	Chi-Tn	Tn-antigen	Lung cancer (in vitro)	[61]
Lipid nanocapsule	Paclitaxel	α-CD44	CD44	Pancreatic cancer (in vitro and in vivo)	[62]
Solid lipid nanoparticle	γ-secretase inhibitor	α-DR5	DR5	Breast cancer (in vitro and in vivo)	[64]
Halloysite nanotube	Docetaxel+ gold	Clone 2D8-E3	Sortilin	Ovarian carcinoma (in vitro)	[66]
PEG-polymer/ prodrug	Camptothecin	α-CD147	CD147	Liver cancer (in vitro)	[67]

**Table 5:** Examples of antibody fragment-functionalized nanoparticles for targeted drug delivery.

Carrier	Drug	Antibody Fragment	Target	Tumor Model	Ref.
Prodrug nanoparticle	DM1	Trastuzumab scFv	HER2	Breast cancer (in vitro and in vivo)	[71]
Mesoporous silica nanoparticle	Doxorubicin	α-HER2 scFv	HER2	Breast cancer (in vitro and in vivo)	[72]
Polystyrene nanoparticle		α-CD44v6 half antibody	CD44	Gastric cancer (in vitro)	[73]
F127 nanomicelle	Niclosamide	α-CD44v6 Fab	CD44	Colorectal cancer (in vitro and in vivo)	[74]
LipoDox^®^	Doxorubicin	α-HER2 scFv + α-mPEG Fab	HER2	Ovarian cancer (in vitro and in vivo)	[75]
LipoDox^®^	Doxorubicin	Ofatumumab + α-mPEG Fab	CD20	B-cell malignancy (in vitro and in vivo)	[76]
mPEG/lecithin nanomicelle	Docetaxel	α-EGFR scFv + α-FAP scFv + α-mPEG Fab	EGFR, FAP	Pancreatic and colorectal cancer (in vitro and in vivo)	[77]

**Table 6:** Examples of peptide-functionalized nanoparticles for targeted drug delivery.

Carrier	Drug	Peptide	Target	Tumor Model	Ref.
Albumin/red cell membranes	Gefitinib	cRGD	α_v_β_3_/α_v_β_5_ integrins	Lung cancer (in vitro and in vivo)	[86]
Lipid nanoparticle	GNA002	cRGD-Arg_6_	α_v_β_3_/α_v_β_5_ integrins + cell penetration	Squamous cell Carcinoma-tongue (in vitro and in vivo)	[87]
Silk fibroin nanoparticle	Curcumin	cRGD	α_v_β_3_/α_v_β_5_ integrins	Different cell models (in vitro)	[88]
Liposome	Doxorubicin	cRGD	α_v_β_3_/α_v_β_5_ integrins	Colon cancer (in vitro and in vivo)	[89]
DSPE/PEG nanoparticle	TQs-PEG4	cRGD	α_v_β_3_/α_v_β_5_ integrins	Breast cancer (in vitro and in vivo)	[90]
Silver sulfide nanoparticle	Doxorubicin	cRGD	α_v_β_3_/α_v_β_5_ integrins	Breast cancer (in vitro and in vivo)	[91]
Gold-iron oxide nanoparticle	Multimodal	cRGD	α_v_β_3_/α_v_β_5_ integrins	Breast cancer (in vitro and in vivo)	[92]
Gallic acid/Fe^3+^ nanoparticle	Doxorubicin, multimodal	cRGD-Platinum prodrug	α_v_β_3_/α_v_β_5_ integrins	Glioblastoma (in vitro and in vivo)	[93]
DSPE nanoparticle	Siponimod	cRGD-PEG	α_v_β_3_/α_v_β_5_ integrins	Breast cancer (in vitro and in vivo)	[94]
PEG-lipid nanoparticle	Wee1 siRNA	cRGD-Arg_8_	α_v_β_3_/α_v_β_5_ integrins + cell penetration	Melanoma (in vitro and in vivo)	[95]
Chitosan nanoparticle	VEGF siRNA	cRGD-albumin	α_v_β_3_/α_v_β_5_ integrins + protective corona	Liver cancer (in vitro and in vivo)	[96]
PLGA nanoparticle	Paclitaxel	iRGD co-administration	α_v_β_3_/α_v_β_5_ integrins + internalization	Colorectal cancer (in vitro and in vivo)	[100]
Fe-metal organic framework	Sorafenib	iRGD co-administration	α_v_β_3_/α_v_β_5_ integrins + internalization	Liver cancer (in vitro and in vivo)	[101]
Polylysine nanoparticle	FGL-1 siRNA PD-L1 siRNA	iRGD co-administration	α_v_β_3_/α_v_β_5_ integrins + internalization	Lewis lung cancer (in vitro and in vivo)	[102]
Mesoporous silica nanoparticle	Doxorubicin	iRGD conjugate	α_v_β_3_/α_v_β_5_ integrins + internalization	Breast cancer (in vitro and in vivo)	[103]
Tetrahedral framework	Doxorubicin	iRGD conjugate	α_v_β_3_/α_v_β_5_ integrins + internalization	Breast cancer (in vitro and in vivo)	[104]
Polymersome	Doxorubicin	iRGD conjugate	α_v_β_3_/α_v_β_5_ integrins + internalization	Breast cancer (in vitro and in vivo)	[105]
DSPE-PEG nanoparticle	Ursolic acid prodrug	iRGD conjugate	α_v_β_3_/α_v_β_5_ integrins + internalization	Gastric cancer (in vitro and in vivo)	[106]
Lipid nanoparticle	CRM1 inhibitor	iRGD conjugate	α_v_β_3_/α_v_β_5_ integrins + internalization	Melanoma (in vitro and in vivo)	[107]
Lipid nanoparticle	Camptothecin Indocyanine green	iRGD conjugate	α_v_β_3_/α_v_β_5_ integrins + internalization	Liver carcinoma (in vitro and in vivo)	[108]
DSPE-PEG nanoparticle	Doxorubicin prodrug	iRGD conjugate	α_v_β_3_/α_v_β_5_ integrins + internalization	Prostate and breast cancer (in vitro)	[109]
Mesoporous silica nanoparticle	Paclitaxel	Angiopep-2	LRP1	Glioma (in vitro and in vivo)	[110]
Hyaluronic acid nanoparticle	Irinotecan	Angiopep-2	LRP1	Glioma (in vitro)	[111]
Caelyx^®^	Doxorubicin	Leptin peptide Lp31	Leptin receptor Ob-R	Colon cancer (in vitro and in vivo)	[112]
Multicomponent nanostructure	Cisplatin Alpelisib Indocyanine green	dYNH	EphA2	Lung cancer (in vitro and in vivo)	[113]
PEG-liposome	Docetaxel	AE147	uPAR	Breast cancer (in vitro and in vivo)	[114]
PEG-dendritic nanomicelle	Paclitaxel	(D-Lys)-LHRH	LHRH receptor	Breast cancer (in vitro and in vivo)	[115]
Prodrug-peptide nanoparticle	Doxorubicin	FRRG	Cathepsin B substrate	Breast cancer (in vitro and in vivo)	[116]
Branched polymer nanoparticle	AZD2281 Pyropheophorbide a	GFLG	Cathepsin B substrate	Breast cancer (in vitro and in vivo)	[117]
Caelyx^®^	Doxorubicin	CGKRK		Breast cancer (in vitro and in vivo)	[118]
Liposome	Curcumin Indocyanine green	GE11 (phage display)		Lung cancer (in vitro)	[119]
Polydopamine nanoparticle	Doxorubicin Phthalo- cyanine	QRH (phage display)		Breast cancer (in vitro and in vivo)	[120]
Polymersome	Doxorubicin	SP94 (phage display)	Cancer cell surface	Liver cancer (in vitro and in vivo)	[121]
DSPE-prodrug nanoparticle	Cisplatin Paclitaxel	Cyclic TMTP1 (bacterial display)	Cancer cell surface	Squamous cell carcinoma-uterus (in vitro and in vivo)	[122]
Graphene oxide-PEG nanoparticle	Doxorubicin	HN-1 (phage display)	Cancer cell surface	Squamous cell carcinoma-oral (in vitro and in vivo)	[123]
Liposome	Doxorubicin Sorafenib	LinTT-1 (phage display)	gC1qR M2 macrophages	Breast cancer (in vitro and in vivo)	[124]
Hyaluronic acid PEG-nanoparticle	miR125b	M2 peptide (phage display)	M2 macrophages	Pancreatic cancer (in vitro and in vivo)	[125]
PEG-prodrug nanoparticle	Irinotecan (SN38)	IELLQAR	E-selectin Endothelial cells	Colon cancer and melanoma (in vitro and in vivo)	[126]
Lipoprotein nanoparticle	Paclitaxel GANT61	tLyP-1	Neuropilin-1 Endothelial cells	Breast cancer (in vitro and in vivo)	[127]

**Table 7:** Examples of protein-functionalized nanoparticles for targeted drug delivery.

Carrier	Drug	Protein	Target	Tumor Model	Ref.
Liposome	Vincristine	Transferrin-mimetic	TfR1	Glioma (in vitro and in vivo)	[129]
PLGA-PEG nanoparticle	Seliciclib	Transferrin-mimetic	TfR1	Different cell models (in vitro)	[130]
Chitosan nanoparticle	Docetaxel Curcumin	Transferrin-mimetic + phenylboronic acid	TfR1	Lung cancer (in vitro and in vivo)	[131]
Carbon-nitride dot	Doxorubicin	Transferrin	TfR1	B-cell malignancy (in vitro and in vivo)	[132]
PEG-albumin nanoparticle	Doxorubicin	Transferrin	TfR1	Breast cancer (in vitro and in vivo)	[133]
Mesoporous silica nanoparticle	Doxorubicin	Transferrin (+ RGD)	TfR1	Different cell models (in vitro)	[134]
Glycyrrhizic acid/PLGA nanoparticle	Piperine	Transferrin	TfR1	Different cell models Breast cancer (in vitro and in vivo)	[135]
PC/DSPE-PEG nanoparticle	Plumbagin	Transferrin	TfR1	Melanoma (in vitro and in vivo)	[136]
Liposome	Erianin	Transferrin	TfR1	Liver cancer (in vitro and in vivo)	[137]
Different formulations	Thymoquinone Gefitinib	Transferrin	TfR1	Lung cancer (in vitro and in vivo)	[138]
Ferritin nanocage	Paclitaxel ERK inhibitor	Ferritin	TfR1	Breast cancer (in vitro)	[140]
Ferritin nanocage	Doxorubicin	Ferritin (+α_2_β_1_ integrin-binding)	TfR1	Glioma (in vitro and in vivo)	[141]
Ferritin nanocage	Paclitaxel	Ferritin (+ tLyP-1 peptide)	TfR1	Breast cancer (in vitro and in vivo)	[142]
Lactoferrin nanoparticle	Docetaxel Celastrol	Lactoferrin	TfR1 and others	Breast cancer (in vitro and in vivo)	[143]
Lactoferrin nanoparticle	Carboplatin Etoposide	Lactoferrin	TfR1 and others	Retinoblastoma (in vitro)	[144]
Ultra-small silica nanoparticle	Doxorubicin	Lactoferrin	TfR1 and others	Glioma (in vitro)	[145]
PEG-liquid crystalline nanoparticle	Imatinib	Lactoferrin	TfR1 and others	Liver cancer (in vitro and in vivo)	[146]
Nanogel	Doxorubicin	Lactoferrin + phenylboronic acid	TfR1 and others	Brain tumors (in vitro and in vivo)	[147]
Iron oxide nanoparticle	Paclitaxel	Folate	Folate receptor	Tumor cell line (in vitro)	[148]
Albumin nanoparticle	Paclitaxel 2-methoxy- estradiol	Folate	Folate receptor	Squamous cell carcinoma- esophagus (in vitro)	[149]
PEG-prodrug nanoparticle	Paclitaxel	Folate	Folate receptor	Breast cancer (in vitro and in vivo)	[150]
Liposome	Paclitaxel Vinorelbine	Folate	Folate receptor	Lewis lung cancer (in vitro and in vivo)	[151]
DSPE-PEG nanoparticle	Doxorubicin Berberine	Folate + hyaluronic acid	Folate receptor	Breast cancer (in vitro and in vivo)	[152]
Polymeric nanoparticle	Paclitaxel Baicalein	Folate	Folate receptor	Breast cancer (in vitro and in vivo)	[153]
PEI-cyclodextrin nanoparticle	Doxorubicin hTERT siRNA	Folate	Folate receptor	Breast cancer (in vitro)	[154]

**Table 8:** Examples of aptamer-functionalized nanoparticles for targeted drug delivery.

Carrier	Drug	Aptamer	Target	Tumor Model	Ref.
Gold nanoparticle	Doxorubicin	By SELEX	PrP^C^	Colon cancer (in vitro)	[159]
PLA-PEG/dextran nanoparticle		A10	PSMA	Prostate cancer (in vitro)	[160]
DSPE-PEG nanoparticle	Paclitaxel + siRNAs	α-PSMA	PSMA	Prostate cancer (in vitro and in vivo)	[161]
Mesoporous polydopamine nanoparticle	Docetaxel	AS1411	Nucleolin	Prostate cancer (in vitro and in vivo)	[163]
Mesoporous silica nanoparticle	Doxorubicin + Tie2 siRNA	AS1411	Nucleolin	Breast cancer (in vitro and in vivo)	[164]
Mesoporous silica/ chitosan nanoparticle	Doxorubicin + α-miR21	AS1411	Nucleolin	Colon cancer (in vitro and in vivo)	[165]
Gold/chitosan nanoparticle	Doxorubicin + paclitaxel	AS1411	Nucleolin	Glioblastoma (in vitro)	[166]
Gold/DNA nanocage	Doxorubicin + VEGF siRNA	AS1411	Nucleolin	Lung cancer (in vitro and in vivo)	[167]
PEG/PAMAM dendrimer nanoparticle	Doxorubicin + HSP70/90 siRNA	AS1411	Nucleolin	Breast cancer (in vitro and in vivo)	[168]
Polymersome	Camptothecin	AS1411	Nucleolin	Colon cancer (in vitro and in vivo)	[169]
PLA-PEI nanomicelle	Camptothecin + survivin shRNA	AS1411	Nucleolin	Colon cancer (in vitro and in vivo)	[170]
Starch nanoparticle	Coumaric acid	AS1411	Nucleolin	Breast cancer (in vitro)	[171]
Nanocapsule + gel	5-FU	AS1411	Nucleolin	Basal carcinoma cells + healthy animals (in vitro and in vivo)	[172]
Gold nanoparticle + gel	C8 + imiquimod	AS1411	Nucleolin	Various tests (in vitro)	[173]
Mesoporous silica nanoparticle	Doxorubicin	α-mucin 1 α-ATP	Mucin 1 ATP	Breast/colon cancer (in vitro and in vivo)	[174]
Iron oxide-pyoverdine nanoparticle	Doxorubicin	α-mucin 1	Mucin 1	Colon cancer (in vitro and in vivo)	[175]
Hydrogel/quantum dot	Paclitaxel + sodium oxamate	α-mucin 1	Mucin 1	Breast cancer (in vitro)	[176]
PEG/PAMAM dendrimer nanoparticle	Gefitinib	α-mucin 1	Mucin 1	Breast cancer (in vitro and in vivo)	[177]
PEG-liposome	Doxorubicin	α-EpCAM	EpCAM	Colon cancer (in vitro and in vivo)	[178]
Iron oxide nanoparticle	Paclitaxel	α-EpCAM	EpCAM	Different cell models (in vitro)	[179]
Polymeric nanomicelle	Temozolomide + idasanutlin	α-CD133	CD133	Glioblastoma (in vitro)	[180]
PAMAM dendrimer nanoparticle	Temozolomide + paclitaxel	B19	CD133	Glioblastoma (in vitro)	[181]
PAMAM dendrimer nanoparticle	Gefitinib + hematoporphyrin	α-EGFR	EGFR	Lung cancer (in vitro)	[182]
PLGA-PEG nanoparticle	Cisplatin +dye	CL4	EGFR	Breast cancer (in vitro and in vivo)	[183]
Mesoporous silica nanoparticle	Doxorubicin	α-HER2	HER2	Breast cancer (in vitro)	[184]

**Table 9:** Examples of sugar-functionalized nanoparticles for targeted drug delivery.

Carrier	Drug	Sugar	Target	Tumor Model	Ref.
PLGA nanoparticle		Hyaluronic acid	CD44, LYVE-1 RHAMM	Different cell models (in vitro)	[188]
Liposome	Oxaliplatin	Hyaluronic acid	CD44, LYVE-1 RHAMM	Colon cancer (in vitro and in vivo)	[189]
Chitosan nanoparticle	Doxorubicin	Hyaluronic acid	CD44, LYVE-1 RHAMM	Breast cancer (in vitro)	[190]
Chitosan nanoparticle	Doxorubicin + miR3a	Hyaluronic acid	CD44, LYVE-1 RHAMM	Breast cancer (in vitro and in vivo)	[191]
Albumin nanoparticle	Doxorubicin	Hyaluronic acid	CD44, LYVE-1 RHAMM	Breast cancer (in vitro)	[192]
Albumin-TPGS nanoparticle	Paclitaxel	Chondroitin sulfate	CD44	Breast cancer (in vitro and in vivo)	[193]
PEI-PLGA nanoparticle	Olaparib	Hyaluronic acid	CD44, LYVE-1 RHAMM	Breast cancer (in vitro and in vivo)	[194]
TPGS-prodrug nanoparticle	Dasatinib	Hyaluronic acid	CD44, LYVE-1 RHAMM	Epithelial tumor cells + healthy animals (in vitro and in vivo)	[195]
mPEG copolymer nanoparticle	Cantharidin	Hyaluronic acid	CD44, LYVE-1 RHAMM	Liver cancer (in vitro and in vivo)	[196]
Prodrug-PLGA nanoparticle	Cabazitaxel + orlistat	Hyaluronic acid	CD44, LYVE-1 RHAMM	Prostate cancer (in vitro and in vivo)	[197]
Silk fibroin nanoparticle	Curcumin + 5-FU	Hyaluronic acid	CD44, LYVE-1 RHAMM	Breast cancer (in vitro and in vivo)	[198]
Poloxamer 407 nanoparticle	Resveratrol + tamoxifen	Hyaluronic acid	CD44, LYVE-1 RHAMM	Breast cancer cells + healthy animals (in vitro and in vivo)	[199]
Polymersome	Doxorubicin + captothecin + α-FOXM1	Hyaluronic acid	CD44, LYVE-1 RHAMM	Lung cancer (in vitro and in vivo)	[200]
Fucoidan nanoparticle	S63845 + venetoclax	Fucoidan	P-selectin	B-cell malignancy (in vitro and in vivo)	[201]
Metal organic framework	Talazoparib + temozolomide	Fucoidan	P-selectin	Colon cancer (in vitro and in vivo)	[202,203]
Ginger lipid nanoparticle	Doxorubicin	Fucoidan	P-selectin	Colon cancer (in vitro and in vivo)	[204]

**Table 10:** Examples of nanoparticles for applications in multidrug resistant tumors.

Carrier	Nucleic Acid	Drug	Natural Compound	Small-Molecule Inhibitor	Targeting Moiety	Ref.
Mesoporous silica nanoparticle	P-gp siRNA					[207]
Prodrug nanoparticle	P-gp siRNA	Paclitaxel				[208]
Chitosan-PEI nanoparticle	P-gp shRNA	Paclitaxel			Folate	[209]
Albumin nanoparticle	P-gp siRNA	Doxorubicin			Cetuximab	[210]
RNA nanoparticle	miR-122	Paclitaxel			Asialoglycoprotein	[211]
Silica/gold nanoparticle	miR-let-7a	Paclitaxel			Hyaluronic acid	[212]
Mesoporous silica nanoparticle	GNC5 siRNA	Doxorubicin			Hyaluronic acid	[213]
Prodrug-PLGA nanoparticle	CCAT2 siRNA	Docetaxel			Transferrin	[214]
Carbon/silica nanoparticle		Doxorubicin		HM30181A	Fucoidan	[216]
Nanomicelle		Paclitaxel		Ritonavir	Hyaluronic acid	[217]
Liposome		Paclitaxel + doxorubicin				[218]
DSPE-PEG nanobubble		Paclitaxel + gemcitabine				[219]
Liposome +hydrogel		Paclitaxel + gemcitabine			PR_b peptide	[221]
Nanocrystal + hydrogel		Paclitaxel + niclosamide				[222]
Lipid nanocapsule		Paclitaxel + salinomycin				[223]
Mesoporous silica nanoparticle		Cisplatin + gemcitabine			α-mucin 1	[224]
PEG-PLGA nanoparticle		Paclitaxel	Curcumin		cRGD	[225]
Polymeric nanoparticle		Paclitaxel	Resveratrol			[226]
PEG-PLGA nanoparticle		Paclitaxel	Etoposide			[227]
Solid lipid nanoparticle		Paclitaxel	Naringenin		cRGD	[228]
PEG-PLGA nanoparticle		Paclitaxel + verteporfin	Combretastatin			[229]
Stealth nanoparticle		Paclitaxel		17-AAG		[230]
Calcium carbonate nanoparticle		Paclitaxel		Gefitinib		[231]
Albumin nanoparticle		Paclitaxel		Carfilzomib		[232]
Copolymer micelle	STAT3 siRNA	Paclitaxel			Hyaluronic acid	[233]
PEI-PLGA nanoparticle	VEGF siRNA	Paclitaxel				[234]
Liposome	Twinfilin 1 siRNA	Paclitaxel				[235]
Polymeric nanoparticle	miR-124	Paclitaxel			Hyaluronic acid	[237]
Lipid nanoparticle	EGFR siRNA	Paclitaxel		Gefitinib	LHRH	[238]

**Table 11:** Examples of strategies adopted to optimize nanovaccine formulations.

Carrier	Antigen(s)	Adjuvant	Route of Administration	Optimized Step	Model	Ref.
PEI-based nanocomplex		CpG	Intratumor injection	Adjuvant formulation	Melanoma	[240]
PEI-PEG nanoparticle	Adpgk M27/M30	CpG	Intratumor injection	Adjuvant formulation	Colon cancer, melanoma	[241]
Lipid-polyGlu nanoparticle	Ovalbumin	Imidazoquinoline	Subcutaneous/ intravenous injection	Adjuvant/antigen coencapsulation	Healthy animals	[242]
PLGA nanoparticle	Ovalbumin	Riboxxim	Intramuscular injection	Adjuvant/antigen coencapsulation	Thymoma, melanoma	[243]
Erythrocyte membrane DSPE-PEG nanoparticle	MAGE-1A + NY-ESO-1 (linker cleaved by cathepsin B)	Not included	Intratumor injection	Inclusion of multiple peptides	Breast carcinoma	[244]
PLGA, PEI-DNA, silica nanoparticle	Cancer cell membranes	CpG	Subcutaneous injection	Inclusion of multiple peptides	Melanoma	[245]
PLGA nanoparticle	Cancer cell membranes	Imiquimod	Intratumor injection	Inclusion of multiple peptides	Breast carcinoma	[246]

**Table 12:** Broadly utilized antigens and examples of corresponding cancer nanovaccines.

Carrier	Antigen(s)	Adjuvant	Model	Ref.
PC7A nanoparticle	Melanoma: Trp1, Tnpo3		Melanoma	[247]
Polyplexes	Melanoma: Trp1	CpG	Melanoma	[248]
Nanomicelle	Melanoma: Reps1	Imidazoquinoline	Melanoma	[249]
Gold nanoparticle	Mucin glycopeptide	α-galactosyl ceramide	MUC1-tumor	[250]
Gold nanoparticle	mucin glycopeptide + ovalbumin	Sigma Adjuvant System or TiterMax Gold	Healthy animals	[251]
Copolymer nanoparticle	Mucin 4β		Healthy animals	[252]
PLGA nanoparticle	NY-ESO-1 peptides	α-galactosyl ceramide	Healthy animals	[253]
Vx3-alumina nanoparticle	Ubiquitinated proteins	STING agonist	Breast cancer	[254]
Silica nanoparticle	E7 GF001 peptide (HPV16)		HPV tumor	[255]
Ultra-small nanoparticle	E7 long peptide (HPV16)	CpG	Head and neck cancer	[256]

**Table 13:** Examples of multimodal cancer nanovaccine.

Carrier	Antigen	Adjuvant	Additional Component	Additional Function	Model	Ref.
Dendrimer nanomicelle	Cancer cell antigens	2-amino imidazole	Chlorin e6	Activation by NIR irradiation	Breast cancer	[257]
PLGA nanoparticle + hydrogel	Ovalbumin	Imiquimod		Activation by ultrasounds	Breast cancer	[259]
Modular nanoparticle	Cancer cell antigens	CpG	Cisplatin	Activation by ROS	Colorectal cancer	[260]
Dextran nanoparticle	Albumin	Imiquimod	Indocyanine green	Photothermal therapy	Thymoma	[261]
Polydopamine nanoparticle + hydrogel	Cancer cell antigens	Imiquimod	Hyaluronic acid Doxorubicin	Photothermal therapy	Breast cancer	[262]
Mesoporous silica nanoparticle	Ovalbumin/cancer cell antigens			Radiotherapy	Liver cancer	[263]

**Table 14:** Examples of nucleic acid-including nanovaccines.

Carrier	Antigen	Adjuvant/Stabilizer	Nucleic Acid	Model	Ref.
DSPE-PEG-PAMAM nanoparticle	Ovalbumin		RNA	Colon cancer, melanoma	[264]
Cationic polymer nanoparticle	Ovalbumin	R848	RNA	Lymphoma, prostate cancer	[265]
PEG-liposome	Total RNA from cancer cells		RNA	Colon cancer	[266]
Calcium phosphate nanoparticle	Ovalbumin	Biphosphonate	DNA	Thymoma	[267]
Calcium phosphate nanoparticle	Ovalbumin	ATP	DNA	Thymic lymphoma	[268]
DNA-RGD nanoparticle	E7 from HPV16	HSP110	DNA	HPV tumor	[269]
Chitosan nanoparticle	Carbonic anhydrase	L-Myc	DNA	Renal cancer	[270]
